# *Staphylococcus aureus* Colonization of the Mouse Gastrointestinal Tract Is Modulated by Wall Teichoic Acid, Capsule, and Surface Proteins

**DOI:** 10.1371/journal.ppat.1005061

**Published:** 2015-07-22

**Authors:** Yoshiki Misawa, Kathryn A. Kelley, Xiaogang Wang, Linhui Wang, Wan Beom Park, Johannes Birtel, David Saslowsky, Jean C. Lee

**Affiliations:** 1 Division of Infectious Diseases, Department of Medicine, Brigham and Women’s Hospital, Harvard Medical School, Boston, Massachusetts, United States of America; 2 Department of Internal Medicine, Seoul National University College of Medicine, Seoul, Republic of Korea; 3 Department of Pediatrics, Boston Children’s Hospital, Harvard Medical School, Boston, Massachusetts, United States of America; University of Tubingen, GERMANY

## Abstract

*Staphylococcus aureus* colonizes the nose, throat, skin, and gastrointestinal (GI) tract of humans. GI carriage of *S*. *aureus* is difficult to eradicate and has been shown to facilitate the transmission of the bacterium among individuals. Although staphylococcal colonization of the GI tract is asymptomatic, it increases the likelihood of infection, particularly skin and soft tissue infections caused by USA300 isolates. We established a mouse model of persistent *S*. *aureus* GI colonization and characterized the impact of selected surface antigens on colonization. In competition experiments, an acapsular mutant colonized better than the parental strain Newman, whereas mutants defective in sortase A and clumping factor A showed impaired ability to colonize the GI tract. Mutants lacking protein A, clumping factor B, poly-N-acetyl glucosamine, or SdrCDE showed no defect in colonization. An *S*. *aureus* wall teichoic acid (WTA) mutant (Δ*tagO*) failed to colonize the mouse nose or GI tract, and the *tagO* and *clfA* mutants showed reduced adherence in vitro to intestinal epithelial cells. The *tagO* mutant was recovered in lower numbers than the wild type strain in the murine stomach and duodenum 1 h after inoculation. This reduced fitness correlated with the in vitro susceptibility of the *tagO* mutant to bile salts, proteases, and a gut-associated defensin. Newman Δ*tagO* showed enhanced susceptibility to autolysis, and an autolysin *(atl) tagO* double mutant abrogated this phenotype. However, the *atl tagO* mutant did not survive better in the mouse GI tract than the *tagO* mutant. Our results indicate that the failure of the *tagO* mutant to colonize the GI tract correlates with its poor adherence and susceptibility to bactericidal factors within the mouse gut, but not to enhanced activity of its major autolysin.

## Author Summary

*Staphylococcus aureus* persistently colonizes ~20% of the human population, and 40–60% of humans are intermittently colonized by this bacterium. The most common reservoir for *S*. *aureus* is the anterior nares, and the incidence of staphylococcal disease in higher in individuals who are colonized. Rectal colonization by *S*. *aureus* isolates, reflecting gastrointestinal (GI) carriage, has recently been recognized as an important reservoir from which person to person transmission occurs. We developed a murine model of *S*. *aureus* GI colonization to investigate bacterial factors that promote staphylococcal colonization of the gut. We identified several surface-associated *S*. *aureus* antigens that modulate colonization of the GI tract and identified a surface glycopolymer (cell wall teichoic acid) as critical for the early steps in colonization. The failure of the teichoic acid mutant to colonize the GI tract can be attributed to its defects in bacterial adherence and to its enhanced susceptibility to mammalian host defenses unique to the gastrointestinal tract. Efforts to develop antimicrobials that target WTA may lead to an overall reduction in asymptomatic colonization by antibiotic-resistant *S*. *aureus* and may impact the incidence of invasive disease.

## Introduction

*Staphylococcus aureus* is a bacterial pathogen that commonly colonizes the nose, skin, and mucosal surfaces of healthy individuals. However, *S*. *aureus* may also cause a variety of superficial and invasive infections in hospitalized patients, as well as in individuals within the community who lack the risk factors commonly associated with nosocomial infections [1,2]. Although the anterior nares are the most common anatomic site of *S*. *aureus* carriage, ~20% of adults are positive for intestinal carriage of *S*. *aureus* [3]. The gastrointestinal (GI) tract has been shown to be a potentially important reservoir for *S*. *aureus* in several clinical studies [4–6]. Although nasal carriage apparently predisposes the host to intestinal carriage, ~37% of intestinal carriers are not positive for *S*. *aureus* nasal colonization [3]. Compared to nasal colonization only, simultaneous nasal and intestinal colonization was associated with a significant increase in the frequency of positive skin cultures [7]. Squier et al. [8] observed that critically ill patients who had both rectal and nasal carriage were significantly more likely to develop staphylococcal infection (40% infection rate) than those with nasal carriage only (18% infection rate).

Patients positive for staphylococcal GI colonization often contaminate their environment with *S*. *aureus* [3,9]. Thus, intestinal carriage may serve as an important reservoir for *S*. *aureus* transmission, contributing to bacterial dissemination and subsequent risk of infection [3]. Faden et al. compared methicillin-resistant *S*. *aureus* (MRSA) nasal and rectal colonization rates in children with staphylococcal skin abscesses and a control group of children without staphylococcal disease [10]. Whereas rates of nasal colonization were equivalent for both groups of children, MRSA was detected significantly more often in the rectum of children with skin abscesses (47%) compared with controls (1%). Moreover, *S*. *aureus* recovered from the abscesses and rectum were identical in 88% of cases, compared with 75% of nasal isolates. Almost all abscess isolates (57/60) were USA300 strains, whereas only 2 of 22 isolates from the control groups were USA300. In a study of HIV-infected men who have sex with men, Szumowski et al. reported that perianal (but not nasal) colonization by MRSA was significantly associated with skin abscess formation [11]. These studies suggest that rectal colonization by *S*. *aureus*, probably reflecting gastrointestinal carriage, is an important reservoir from which person to person transmission occurs.

Host factors that facilitate staphylococcal colonization of the GI tract are poorly understood. Intestinal carriage occurs at a high frequency within the first six months of life, after which the incidence drops [3]. Additional factors, such as decreased stomach acidity, antibiotics that disrupt the indigenous microbiota, or the administration of cyclophosphamide or prednisone, may also influence acquisition of *S*. *aureus* in the human GI tract [4–6].

Whereas staphylococcal factors that promote GI colonization have not been reported, several independent investigations have identified surface antigens that impact *S*. *aureus* nasal colonization in rodent models. Mutants deficient in either clumping factor B or cell wall teichoic acid (WTA) showed reduced nasal carriage in experimentally inoculated rats or mice [12,13], and clumping factor B promoted nasal colonization of humans [14].

In this investigation, we sought to develop and characterize a reliable murine model of *S*. *aureus* GI carriage to better understand its relevance as a risk factor for subsequent infection and its potential for transmission and spread of this pathogen. *S*. *aureus* antigens important for nasal colonization were assessed to determine whether they might also play a role in colonization of the GI tract. We identified several surface-associated *S*. *aureus* antigens that modulated colonization of the GI tract and identified WTA as critical for the early steps in colonization. The failure of the WTA mutant (*ΔtagO)* to colonize the GI tract correlated with its defects in bacterial adherence and greater susceptibility to antimicrobial factors within the mouse gut.

## Results

### Establishment of an *S*. *aureus* gastrointestinal colonization model in mice

The mouse intestinal tract is comprised of diverse commensal bacteria, including Bacteroides, Clostridia, segmented filamentous bacteria, members of the Enterobacteriaceae, Lactobacilli, and Enterococci [15,16]. These normal flora provide some protection against invading pathogens, as evidenced by the fact that we were unable to establish stable (≥ 1 week) GI colonization by *S*. *aureus* in conventional mice in the absence of selective antibiotic pressure **(S1 Fig).** In previous studies, we maintained stable nasal colonization of mice with *S*. *aureus* by supplementing their drinking water with streptomycin (Sm) and inoculating with Sm-resistant (Sm^r^) staphylococcal isolates [12,17], and a similar approach was taken here. Fecal cultures performed on mice prior to inoculation were negative for Sm^r^ *S*. *aureus* (lower limit of detection ~3 log CFU *S*. *aureus/*g stool). Awake mice maintained on Sm water and administered intranasal inocula of Sm^r^ *S*. *aureus* Newman ranging from 8 x 10^8^ to 2 x 10^5^ CFU showed stable colonization of the GI tract for at least 3 wks (**Table 1**). Recovery of Sm^r^ *S*. *aureus* Newman from the stool cultures consistently averaged ~10^5^ CFU/g stool. Differences in GI colonization of the WT strain were not observed when we compared inoculation by the intranasal route with inoculation by oral gavage. A subset of animals were given Sm water during week 1 and then given regular drinking water thereafter. These mice maintained GI colonization for at least 3 weeks at levels (~10^5^ CFU/g stool) similar to those of mice maintained for 3 wks on Sm water (**S1 Fig**).

**Table 1:** Quantitative stool cultures from mice (n = 4–15 per group) that were inoculated with Sm^r^ *S*. *aureus* strain Newman on day zero.

Inoculum (CFU/mouse)	Log CFU *S*. *aureus* Newman/g stool ± SEM at indicated time point ^a^			
	Pre-inoculation	Day 7	Day 14	Day 21
8.2 x 10^8^	<3.0	5.63 ± 0.11	5.10 ± 0.36	5.67 ± 0.27
2.7 x 10^8^	<3.0	5.26 ± 0.20	5.16 ± 0.23	5.15 ± 0.24
1.9 x 10^7^	<3.0	5.57 ± 0.21	4.59 ± 0 62	5.89 ± 0.27
1.9 x 10^5^	<3.0	5.86 ± 0.19	4.68 ± 0.41	5.11 ± 0.37
1.9 x 10^3^	<3.0	3.86 ± 0.46	4.00 ± 0.33	4.00 ± 0.49

^a^ The lower limit of detection by culture is ~3.0 log CFU *S*. *aureus*/g stool.

To determine where *S*. *aureus* resided in the GI tract of conventional mice, we inoculated animals with ~10^9^ CFU of *S*. *aureus*, and euthanized the animals on days 1, 2, 4 and 7. Segments of the GI tract were removed and cultured quantitatively. The colonized staphylococcal density in the conventional mice was quite variable in the small intestine with the highest being observed in the distal segment of the small intestine (1.3 x 10^5^ CFU/g tissue), cecum (3.9 x 10^5^ CFU/g tissue) and colon (3.2 x 10^5^ CFU/g).

To measure the impact of the commensal GI flora on *S*. *aureus* colonization, germfree Swiss Webster mice (n = 8) were inoculated with ~10^9^ CFU of *S*. *aureus*, and quantitative stool cultures were performed on days 1, 5, 7, 12, and 14. In contrast to conventional mice, germfree mice were readily colonized by *S*. *aureus* as early as day 1 without the need for Sm drinking water. Quantitative cultures yielded concentrations ranging from 1 x 10^7^ to 9.7 x 10^8^ CFU/g stool over the course of the two-week time period. Four to five mice were euthanized on day 7 or 14, and segments of the GI tract were removed, homogenized, and cultured quantitatively. Whereas *S*. *aureus* was detected throughout the GI tract at concentrations >10^4^ cfu/g, significantly higher staphylococcal densities were achieved in the distal portions of the GI tract (**Table 2**). These findings corroborated those that we found in conventional mice, i.e., the highest bacterial burdens were localized to the mouse cecum and colon.

**Table 2:** Quantitative cultures of GI segments from germ-free mice inoculated with *S*. *aureus* and evaluated on days 7 or 14.

Sample	*S*. *aureus* Log CFU/g sample ± SEM	
	Day 7 ^a^	Day 14 ^b^
Duodenum	6.20 ± 0.71 ^c^	6.35 ± 0.21 ^d^
Jejunum	6.17 ± 0.43 ^c^	6.65 ± 0.25 ^d^
Ileum	7.41 ± 0.07	7.09 ± 0.21 ^e^
Cecum	7.65 ± 0.09	8.26 ± 0.09
Colon	8.26 ± 0.07	8.73 ± 0.09
Stool	8.03 ± 0.11	8.56 ± 0.05

^a^ *P* = 0.0024 by one-way ANOVA.

^b^ *P <* 0.0001 by one-way ANOVA.

^c^ On day 7, the *S*. *aureus* counts in the duodenum and jejunum were significantly less than those in the ileum (*), cecum (*), and colon (***).

^d^ On day 14, the counts in the duodenum were significantly less than those in the ileum (**), and the counts in the duodenum and the jejunum were less than those in the cecum (****), and colon (****).

^e^ On day 14, the counts in the ileum were less than those in the cecum (***) and colon (****).

*, *P* <0.05; **, *P* <0.01, ***, *P* <0.001, ****, *P* <0.0001.

### Bacterial factors that promote colonization of the GI tract

**Fig 1:**
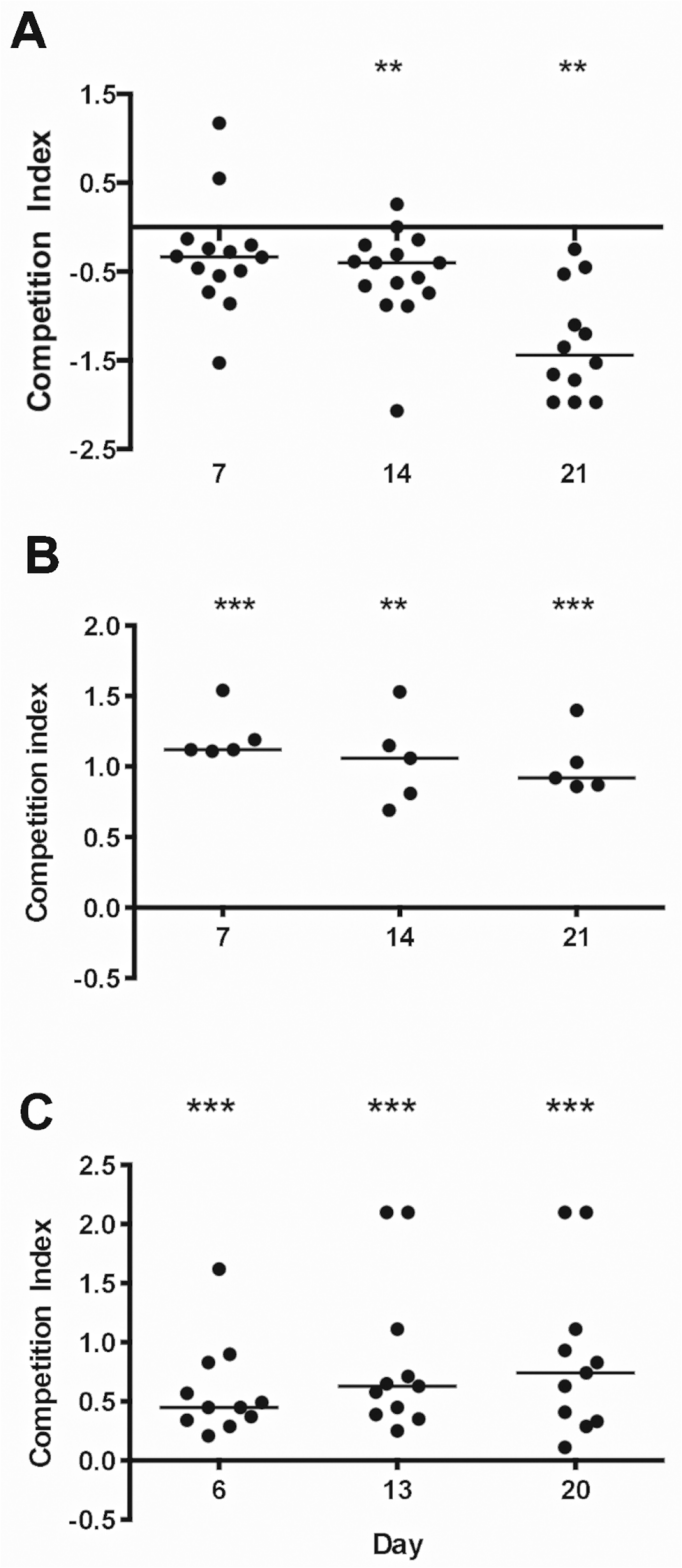
*S*. *aureus* factors that impact colonization of the murine GI tract. **A)** The Newman serotype 5 capsule-negative mutant showed a competitive fitness advantage relative to the wild type Newman capsule-positive strain. **B)** A sortase A mutant and **C)** a clumping factor A (ClfA) mutant showed a competitive defect compared to the parental strain Newman. The competitive index (CI) was defined as the log_10_ output ratio/ input ratio. A CI <0 indicates a mutant with a colonization advantage over the WT strain, and a CI >0 indicates a mutant with a colonization disadvantage compared to the WT. Horizontal bars represent the median competitive index for groups of 5–15 mice. *P*-values were determined by Wilcoxon signed-rank test. *P < 0.05; ** *P* < 0.01; *** *P* < 0.001.

Previous studies revealed that *S*. *aureus* mutants deficient in sortase (Δ*srtA;* affects all cell wall anchored proteins), clumping factor B, the serotype 5 capsule, and WTA were defective in their ability to colonize the nasal cavity of rodents [12,13,17,18]. Evaluation of microbial fitness for colonization of the GI tract is often performed in the context of competition experiments [19–21]. When we evaluated the relative fitness of *S*. *aureus* mutants vs. the parental strain, we observed that the acapsular Newman *cap5G* mutant showed enhanced fitness, colonizing the mouse GI tract in numbers greater than that of the parental type 5 capsule positive strain (**Fig 1**). The sortase A mutant and a clumping factor A (*clfA*) mutant showed impaired fitness in vivo, since both were out-competed by the wild type (WT) strain Newman (**Fig 1**). To validate the latter finding, we repeated the competition experiment between strain Newman and its *clfA* mutant with inoculation by oral gavage. Similar to our initial findings, the *clfA* mutant again showed a significant colonization defect between 7 and 21 days after inoculation **(S2 Fig).** Newman Δ*ica*, Newman Δ*clfB*, Newman Δ*spa*, and Newman Δ*sdrCDE* showed no colonization defect (**S3 Fig**).

When we inoculated separate groups of 5 to 10 mice with the parental strain Newman or isogenic mutants lacking the serotype 5 capsule, sortase, or ClfA, we observed no differences between the colonization levels achieved by the parental or mutant strains (**S4 Fig**). Thus, the colonization defects of these three mutants were only evident as measured by in vivo competition experiments. Likewise, a mutant defective in beta hemolysin (Hlb) colonized the mouse gut at levels consistent with the respective parental strains (**S4 Fig**).

**Fig 2:**
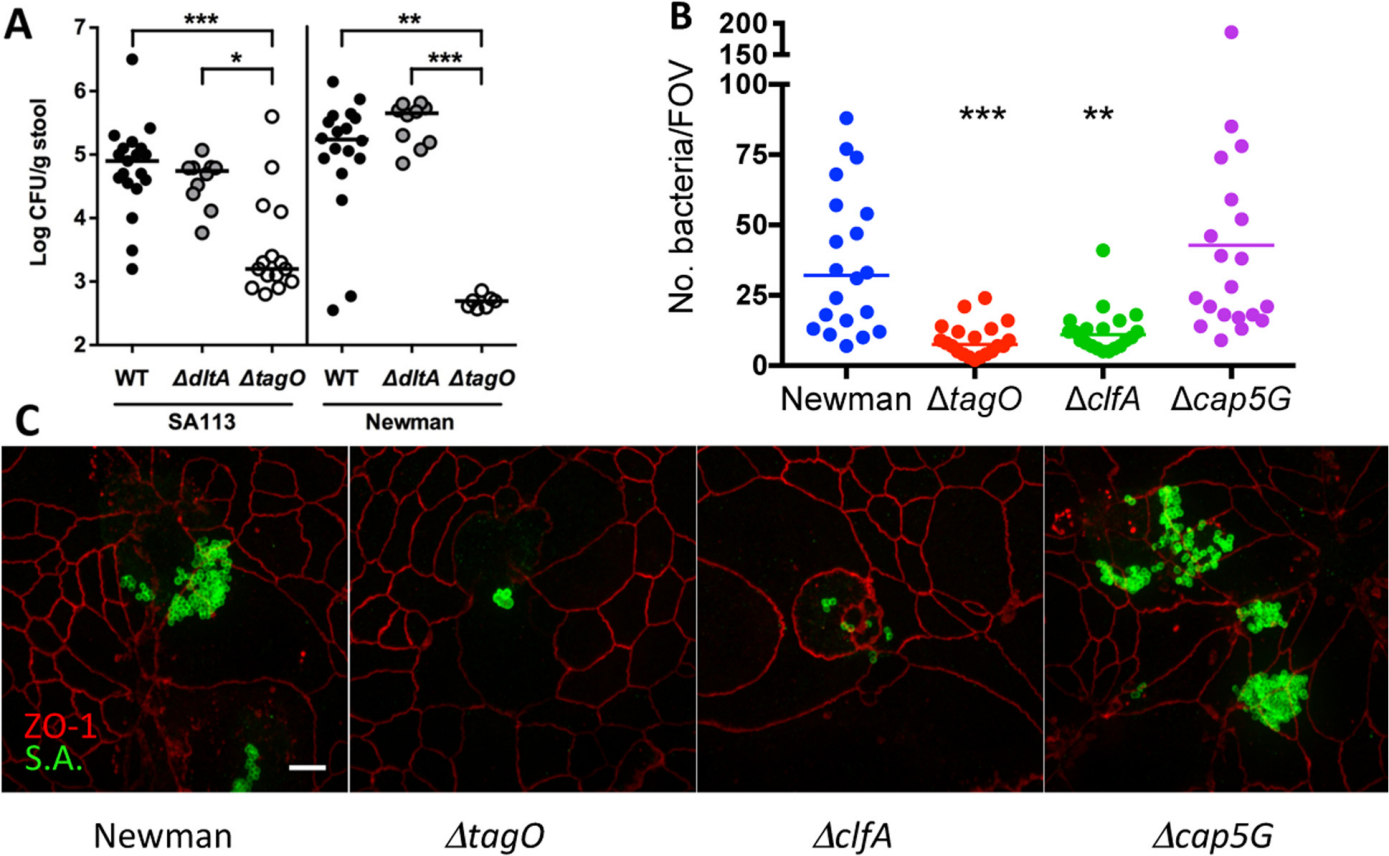
Effects of WTA on *S. aureus* GI colonization and the influence of surface antigens on in vitro adherence of *S. aureus* to T84 cells. **A)** Fecal cultures were performed 7 d after bacterial inoculation with 10^9^ CFU *S. aureus*. Each dot indicates the CFU *S. aureus*/g stool for a single mouse, and the median of each group of animals is indicated by a horizontal line. The lower limit of detection by culture was ~2.5 log CFU/g stool. *P*-values were determined by Kruskal-Wallis test with Dunn’s multiple comparison test. **B)** At an MOI of 100, Newman Δ*tagO* and Δ*clfA* were less adherent in vitro to T84 intestinal epithelial cells than WT strain Newman, as measured by the number of adherent *S. aureus* per field of view (FOV). * *P* < 0.05; ** *P* < 0.01; *** *P* < 0.001. **C)** The relative adherence of strain Newman and its isogenic mutants lacking WTA (Δ*tagO*), ClfA, or capsular polysaccharide (Δ*cap5G*) to T84 cells in vitro was viewed by confocal microscopy. Immunostaining was performed with mouse anti-ZO-1 (red) to visualize the T-84 apical tight junctions and *S. aureus* rabbit antiserum to identify the bacteria (green). Scale bar, 10 microns.

Weidenmaier et al. reported that a *tagO* mutant failed to colonize the nasal cavity of cotton rats [13]. To elucidate the role of WTA in GI colonization, separate groups of mice were inoculated with *S*. *aureus* SA113 or Newman or their isogenic *tagO* or *dltA* mutants. Both SA113 Δ*tagO* and Newman Δ*tagO* showed a significant (*P* <0.01) reduction in GI colonization as early as one week after intranasal inoculation compared to the parental strain **(Fig 2A**), despite the fact that the growth of the mutant in vitro was comparable to that of the parental strain [22]. To confirm these findings, additional groups of mice were inoculated by oral gavage with strain Newman or its *tagO* mutant, and GI colonization was monitored up to 72 h. By 24 h after inoculation, there were significantly fewer Newman *ΔtagO* recovered from the stools of mice inoculated with Newman Δ*tagO* compared to the WT strain **(S5 Fig).** These data suggest that the WTA polymer is a critical determinant for colonization of the GI tract by *S*. *aureus*. To determine whether modifications of the WTA backbone affect GI colonization, we inoculated mice with *dltA* mutants of strain SA113 or Newman, which lack the ester-linked alanine substituent on staphylococcal teichoic acid. The *dltA* mutants showed no colonization defect **(Fig 2A).** Similarly, a Δ*tarM* Δ*tarS* double mutant of *S*. *aureus* RN4220, which lacks alpha- and beta-O-linked GlcNAc modifications of WTA, colonized mice at levels similar to the WT strain **(S6 Fig)**.

WTA has been reported to promote staphylococcal adherence to human nasal epithelial cells and endothelial cells [13,23]. As a result, we compared the relative in vitro adherence of strains Newman and Newman Δ*tagO* to T84 human intestinal epithelial cells. As shown in **Fig 2B and 2C**, strain Newman showed significantly (*P* = 0.0009) greater adherence to the T84 cells than did its Δ*tagO* mutant. Likewise, the *clfA* mutant, which showed an in vivo fitness defect (**Fig 1C**), also showed impaired adherence to T84 cells (**Fig 2B and 2C**; *P* = 0031). The acapsular *cap5G* mutant, on the other hand, showed a modest increase in overall adherence to the epithelial cells, consistent with previous studies showing that the capsule can mask adhesins that mediate attachment to mammalian cells [24,25].

### Competition between Newman and Newman Δ*tagO* within the GI tract

**Fig 3:**
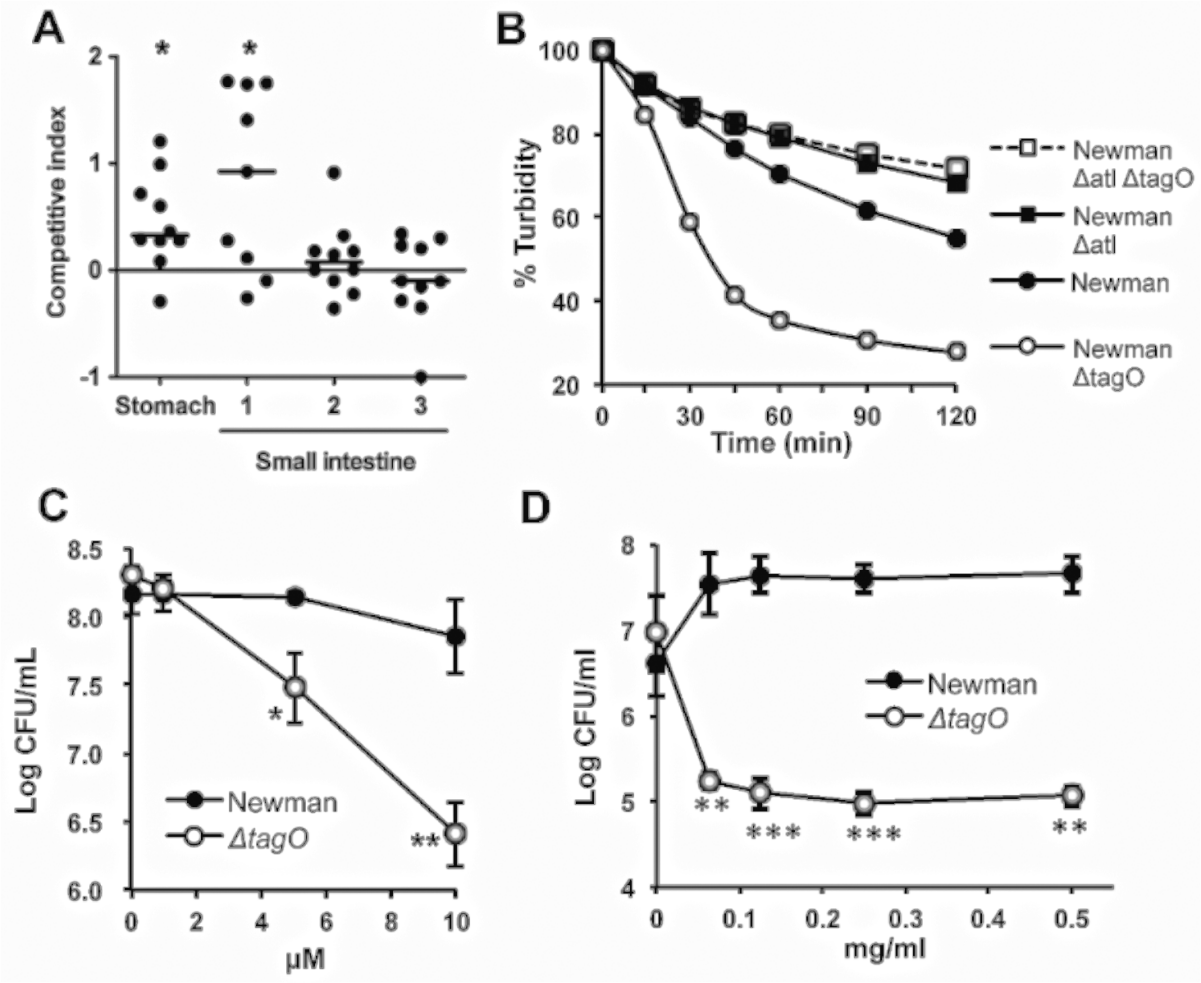
The reduced GI fitness of the Δ*tagO* mutant correlates with its susceptibility to bile, defensins, and proteases. **A)** Mice were inoculated with a 1:1 mixture of the WT and Δ*tagO* mutant. After 1 h segments of the gut were homogenized and cultured quantitatively. The average *S*. *aureus* bacterial burden in the stomach was 3.2 x 10^6^ CFU/g, and the first three segments of the small intestine yielded *S*. *aureus* concentrations of 2.6 x 10^5^, 5.3 x 10^5^ and 3.4 x 10^7^ CFU/g, respectively. Horizontal bars represent the median competitive index for groups of 9–10 mice. *P* values were determined by Wilcoxon signed-rank test. **B)** *S*. *aureus* suspensions were exposed in vitro to bile salts (0.075% sodium deoxycholate), and quantitative cultures were performed to evaluate bacterial survival compared with samples lacking deoxycholate. Data are representative of 3 separate determinations. **C)** *S*. *aureus* were exposed for 2 h to increasing concentrations of α-defensin 5. The data are means ± SEM of 3 experiments. **D)** *S*. *aureus* at 1 x 10^6^ CFU/ml were incubated at 37°C in the presence of increasing concentrations of proteinase K, and bacterial viability was measured after 24 h. The data are means ± SEM of 3 independent experiments. *P*-values were determined by the Student t-test. *P < 0.05, **P < 0.01, ***P < 0.001.

To determine whether a WTA-deficient mutant was competent for transit through the stomach and duodenum to establish a colonization niche in the intestines, we performed several short-term in vivo experiments. Mice were inoculated with either strain Newman or the Newman *tagO* mutant and euthanized after 1 h. Recovery of *S*. *aureus* by quantitative cultures of the stomach and four different segments of the GI tract were quite variable among different mice. For this reason, we performed subsequent experiments in a competition format wherein mice were inoculated with a 1:1 mixture of Newman and Newman Δ*tagO*. A control in vitro competition experiment whereby Newman and its *tagO* mutant were cultured together for 1 h showed a CI of 0. Strain Newman and its *tagO* isogenic mutant were co-inoculated into mice, and after 1 h segments of the gut were homogenized and cultured quantitatively. The Newman Δ*tagO* mutant exhibited a substantial fitness defect in vivo with median CIs of 0.33 and 0.92 in the murine stomach and duodenum, respectively (*P* < 0.05) **(Fig 3A).** Few *S*. *aureu*s colonies were recovered from the fourth segment of the small intestine, the cecum, or the colon, and consequently these data were not analyzed further. Our findings indicate that the Δ*tagO* mutant is significantly impaired in surviving transit through the mouse stomach and duodenum, and that its failure to colonize the GI tract may reflect its inherent susceptibility to antimicrobial factors in the gut.

### Relative susceptibility of the Δ*tagO* mutant to antibacterial factors in the mouse GI tract

Because the *tagO* mutant survived poorly in vivo after inoculation, we hypothesized that Newman Δ*tagO* might be more susceptible than the WT strain to bile salts or low pH. As shown in **Fig 3B**, the *tagO* mutant was more susceptible than strain Newman to killing by bile salts (0.075% sodium deoxycholate), and the killing was rapid (~1 h). Because the concentration of bile salts in hepatic bile can reach concentrations as high as 1.66% [26], the enhanced susceptibility of the *tagO* mutant may contribute to its poor recovery from the gut after only 1 h in vivo. In contrast, both the parental and mutant strains were killed under low pH conditions (glycine buffer, pH 3.0–3.6), and no differences in their relative susceptibilities to acidic conditions were observed (**S7 Fig**).

The antimicrobial peptide α-defensin 5, secreted by mammalian Paneth cells, plays a role in the intestinal host defense against bacterial pathogens. Furthermore, α-defensins demonstrate homeostatic control of the host commensal microbiota [27]. Our in vitro studies indicated that the Newman Δ*tagO* mutant was killed to a significantly greater extent than the WT strain after a 2 h exposure to α-defensin 5 concentrations ranging from 5–10 μM **(Fig 3C),** which are far below the 14–70 μM concentrations found in the human intestinal lumen [28].

Bacteria transiting the GI tract are also exposed to proteolytic enzymes. Although we could not demonstrate consistent differences in susceptibility during short-term exposures to proteases (**S8 Fig**), the *tagO* mutant was more sensitive than the parental strain Newman to overnight treatments with proteinase K **(Fig 3D)**, pepsin (at low pH) and trypsin (**S8 Fig**).

### Bactericidal activity of staphylococcal supernatants for Newman Δ*tagO*

**Fig 4:**
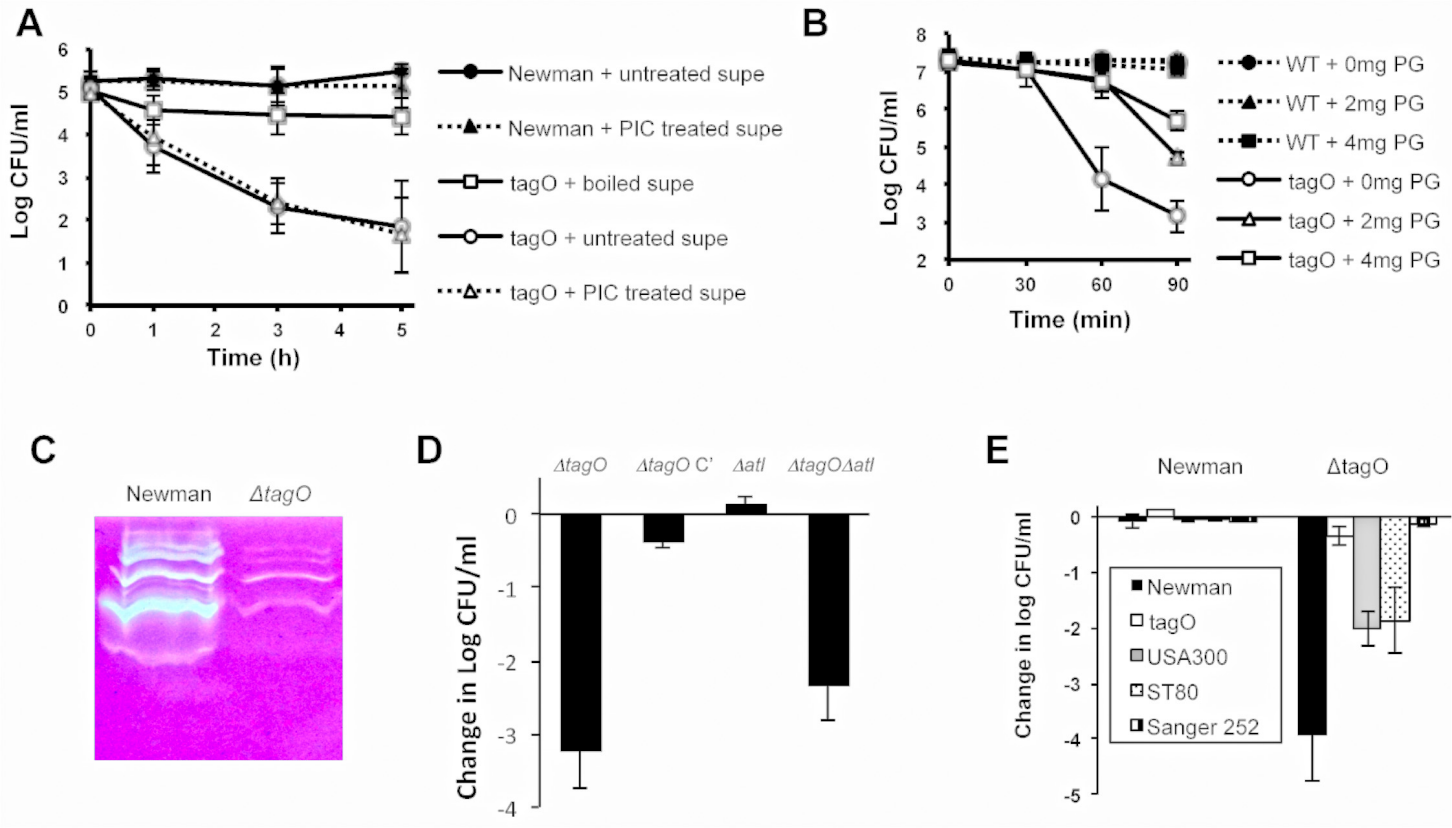
Culture supernatants of WT *S*. *aureus* were bactericidal for Newman *ΔtagO*. **A)** Newman supernatants (untreated), boiled, or supplemented with a protease inhibitor cocktail (PIC) were inoculated with Newman or Newman Δ*tagO* and incubated at 37°C. Bactericidal activity was measured by quantitative cultures at the indicated time points. **B)** Newman culture supernatants were supplemented with 0, 2, or 4 mg peptidoglycan and inoculated with either Newman or Newman Δ*tagO*. CFU/ml determinations were performed to evaluate bacterial viability. The data represent means ± SEM of 3 independent experiments. **C)** Zymogram of Newman and Δ*tagO* culture supernatants. The samples were concentrated and adjusted to equivalent protein concentrations before electrophoresis. **D)** Newman mutants were inoculated into filter-sterilized Newman culture supernatants, and bactericidal activity was measured after 3 h. Δ*tagO* C’ carried pRB*tagO*, which restored WTA production to the *tagO* mutant. **E)** Newman or Newman Δ*tagO* was added to culture supernatants of *S*. *aureus* Newman, Δ*tagO*, ST80, or Sanger 252, and bactericidal activity was measured at 3 h. The data represent means ± SEM of 3–4 experiments.

As noted above, when 10^9^ CFU of Newman and its *tagO* mutant were incubated together in vitro for 1 h at 37°C in tryptic soy broth (TSB; Difo, Sparks, MD), the competitive index was 0, i.e., both strains were recovered in equivalent numbers. However, if the two strains were incubated together overnight in TSB, the *tagO* mutant could not be recovered from the culture (>10^7^ reduction in CFU compared to strain Newman). Similar experiments performed with WT strain SA113 and its *tagO* mutant yielded comparable results (~10^5^ reduction in the SA113 Δ*tagO* CFU compared with SA113). Filter-sterilized culture supernatants of *S*. *aureus* Newman (but not culture supernatants from the *tagO* mutant) also showed bactericidal activity toward Newman Δ*tagO* within 1 to 3 h of incubation **(Fig 4A and 4B).** The bactericidal activity of the Newman culture supernatant was lost if it was boiled for 5 min prior to inoculation with Newman Δ*tagO* **(Fig 4A)**. In contrast, the addition of a protease inhibitor cocktail to the supernatant had no effect on its killing activity **(Fig 4A)**. Consistent with this finding, we observed no differences in the protease activity of culture supernatants derived from the WT or the Δ*tagO* mutant strain when measured on casein agar plates, azocasein assays, or the PDQ protease assay [Athena Environmental Sciences, Inc., Baltimore, MD] (**S9 Fig**). Peptidoglycan fragments added to filter-sterilized culture supernatants of strain Newman inhibited killing of the Δ*tagO* mutant in a dose dependent manner **(Fig 4B).** This finding suggested that autolysins present in the Newman culture supernatant might be lysing the *tagO* mutant. Indeed, zymogram analysis revealed that the Newman culture supernatant showed greater autolytic activity than that of the *tagO* mutant **(Fig 4C).** That the bactericidal effect on the WTA mutant was due to exogenous autolytic activity was supported by the observation that both the *tagO* and an autolysin (*atl*) *tagO* double mutant showed a >2-log reduction in CFU/ml following a 3-h exposure to the Newman culture supernatant **(Fig 4D)**. In contrast, neither strain that produced WTA (the *atl* mutant nor Newman Δ*tagO* complemented with a plasmid carrying the cloned *tagO* gene [Δ*tagO* C’]) were killed after incubation with the Newman supernatant. The susceptibility of the *tagO* mutant to the bactericidal activity of culture supernatants was not limited to strain Newman, since Newman Δ*tagO* was also killed by supernatants harvested from *S*. *aureus* strains USA300 LAC and ST80, but not from Sanger 252 **(Fig 4E)**. None of the culture supernatants were bactericidal against the parental strain Newman.

**Fig 5:**
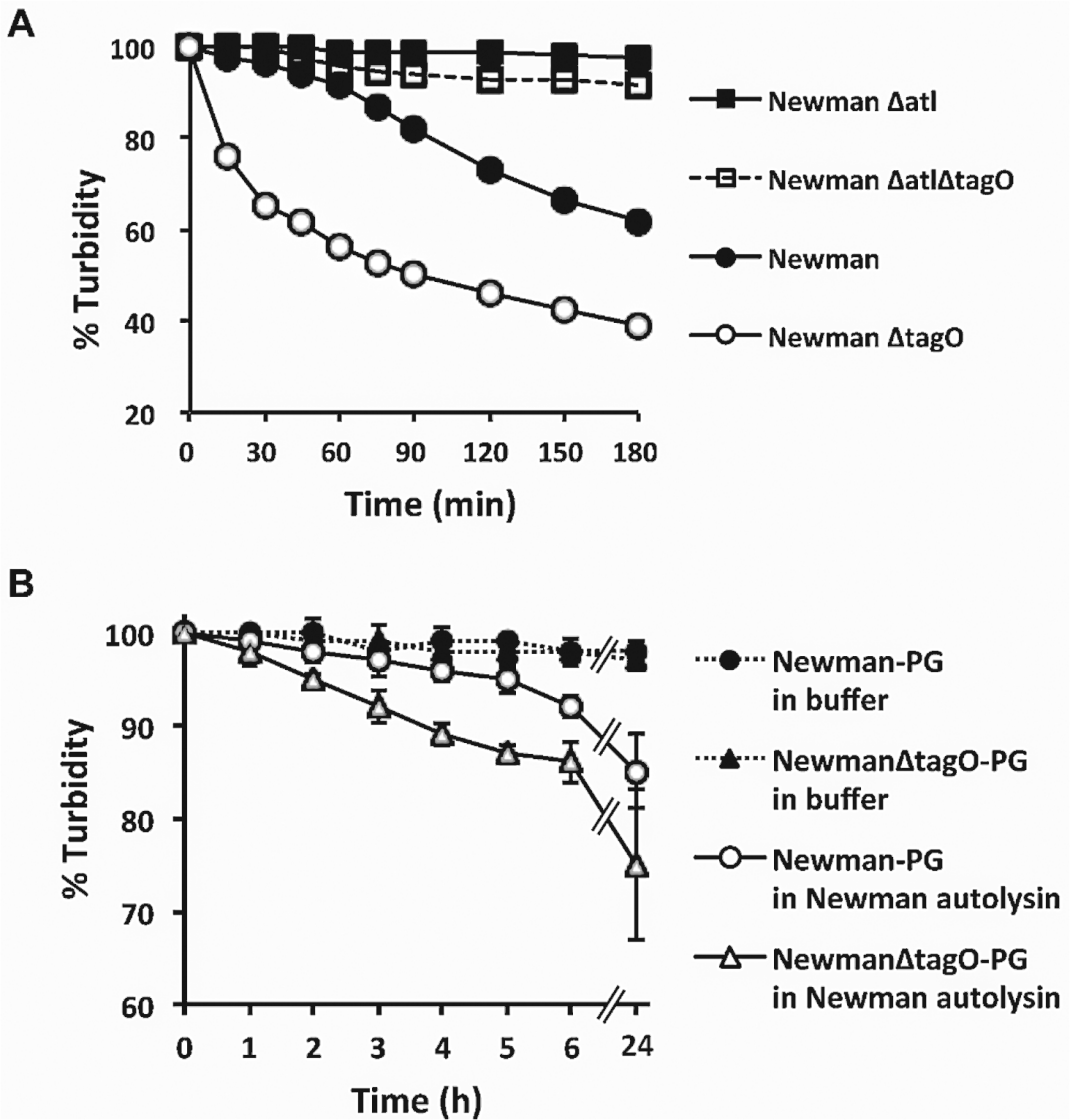
The relative susceptibilities of *S*. *aureus* strains and their peptidoglycan preparations to autolysis. **A)** Triton X-100 induced autolysis of *S*. *aureus* Newman, Δ*tagO*, *Δatl*, and Δ*tagO*Δ*atl* mutants. The results shown are representative of 3 independent determinations. **B)** An *S*. *aureus* Newman autolysin extract (25 μg protein/ml) was prepared and incubated with peptidoglycan isolated from Newman and Newman Δ*tagO*. The data represent means ± SEM of 3 independent experiments. Lysis was expressed as a percentage of the OD_580 nm_ at time zero.

Koprivnjak et al. reported that *S*. *aureus* SA113 Δ*tagO* was more sensitive than the WT strain to Triton X-100 at concentrations >0.1% [29]. We observed that Newman Δ*tagO* also was hypersusceptible to Triton X-100 mediated bacterial lysis at concentrations as low as 0.05% **(Fig 5A).** As expected, a Newman *atl* mutant and an *atl tagO* double mutant were resistant to Triton X-100 induced autolysis **(Fig 5A).** Because factors that stimulate autolytic activity include detergents, proteolytic enzymes, and cationic peptides [30], we hypothesized that the Newman Δ*tagO* might be more sensitive than the WT strain to factors that promote staphylococcal autolysis. This hypothesis is consistent with the observation made by Atilano et al [31] that peptidoglycan prepared from a Δ*tagO* mutant shows reduced cross-linking compared to the WT strain. Autolysin extracts from strain Newman and Newman Δ*tagO* showed similar lytic activity toward peptidoglycan prepared from the strain Lafferty (**S10 Fig**). However, peptidoglycan prepared from the Newman Δ*tagO* mutant was more susceptible to Newman autolysin extracts than a peptidoglycan preparation from the parental strain Newman **(Fig 5B).**

#### GI colonization by a WTA mutant resistant to autolysis (Δ*atl* Δ*tagO*)

**Fig 6:**
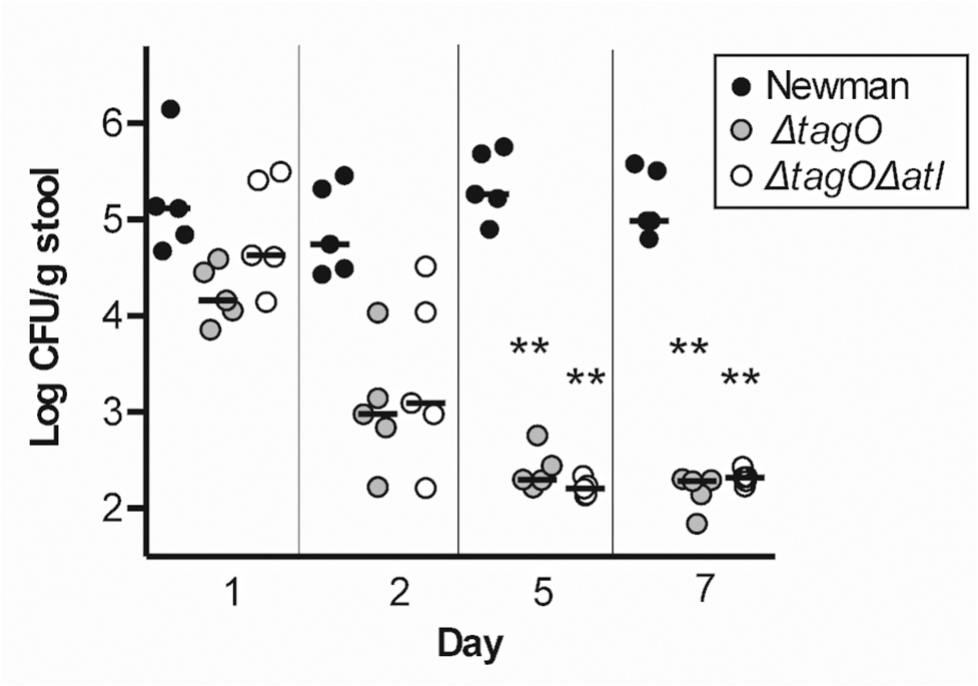
Both Newman Δ*tagO* and Newman Δ*atl* Δ*tagO* fail to persistently colonize the mouse GI tract. Mice were inoculated on day 0 with Newman, Newman Δ*tagO*, or Newman Δ*atl* Δ*tagO*, and stool samples were cultured quantitatively 1, 2, 5, and 7 days after inoculation. Each symbol represents a stool culture from an individual mouse. *P* values were determined by Wilcoxon signed-rank test. **P < 0.01.

To clarify the relationship between the defect in GI colonization of the *tagO* mutant and its susceptibility to autolysis, we inoculated separate groups of mice with Newman, Newman Δ*atl*, Newman Δ*tagO*, or an *atl tagO* double mutant; GI colonization was assessed up to 14 days after inoculation. The Newman Δ*atl* mutant colonized the mouse GI tract as well as the WT strain, but the Δ*tagO* and the Δ*atl* Δ*tagO* double mutant failed to colonize (**S11 Fig**). When the experiment was repeated, we observed that both the *tagO* and the *atl tagO* double mutant were recovered in reduced numbers from the stool as early as two days after inoculation, and by day five neither mutant could be detected in the murine stool cultures (**Fig 6**). Similar results were obtained in mice inoculated by oral gavage (**S5 Fig**). In a competition format, four mice were inoculated with a 1:1 mixture of Newman and the Newman double mutant and euthanized for quantitative cultures after 1 h. Like the *tagO* mutant, Newman Δ*atl* Δ*tagO* exhibited a substantial fitness defect in vivo with a median CI of 1.9 in the stomach and CIs ranging from 0.98 to 1.6 in the proximal small intestine. These results indicate that the failure of *S*. *aureus* lacking WTA to colonize the GI tract is not explained by its enhanced autolytic response (lacking in the double mutant), but instead correlates with its poor adherence and susceptibility to bactericidal factors within the mouse gut.

## Discussion

In addition to its niche within the nares, in the throat, and on the skin of humans, *S*. *aureus* may also colonize the GI tract of ~20% of otherwise healthy individuals [3]. While no disease is clearly associated with GI colonization by staphylococci, it serves as a source of recolonization after eradication of nasal colonization. In a hospital setting or in the community, fecal contamination associated with diarrhea or incontinence could contaminate the environment and facilitate transmission among individuals. As such, the GI tract is an under-appreciated reservoir for methicillin-resistant and-sensitive strains of *S*. *aureus*. Among patients in an intensive care unit or transplant unit, those positive for both rectal and nasal carriage of *S*. *aureus* were more likely to develop a staphylococcal infection than those with nasal carriage alone [8]. Other reports have similarly suggested that GI carriage is an important risk factor for *S*. *aureus* infections [3,4,9,10]. Importantly, staphylococcal skin and soft tissue infections are linked to rectal, but not nasal, colonization by MRSA strains in children [10]. Molecular typing experiments indicated that rectal, nares, and infecting isolates (including blood isolates) were clonally identical in 82% of the patients with S. *aureus* infections [8]. A comprehensive understanding of the factors involved in asymptomatic carriage by *S*. *aureus* in humans is critical to our ability to control the incidence of infection.

Kernbauer et al. [32] recently reported that mice challenged intravenously with *S*. *aureus* develop staphylococcal colonization of the GI tract, and that fecal shedding resulted in *S*. *aureus* transmission to cohoused naïve mice. They noted that GI colonization resulted in no obvious signs of infectious abscesses or inflammation. In our studies, healthy mice inoculated intranasally developed persistent *S*. *aureus* GI colonization only when they were administered Sm in their drinking water for at least one week. This suggests that transient suppression of the indigenous intestinal flora may reduce bacterial interference and created an environment amenable to stable *S*. *aureus* colonization. The concentration of *S*. *aureus* in the cecum, colon, and stool was not high in conventional mice, achieving levels ~10^5^ CFU/g, very similar to those reported by Kernbauer et al. [32]. When germ-free mice that lacked a competing indigenous flora were inoculated with *S*. *aureus*, the concentrations in the intestinal contents and stool rose to ~10^8^ CFU/g.

To investigate bacterial factors that impact GI colonization, we assessed colonization of mice inoculated with a single bacterial strain, and we also performed competition experiments between WT and mutant *S*. *aureus* strains. A Newman type 5 capsule-negative mutant was able to persist in the gut in higher numbers than the WT encapsulated parental strain in competition experiments, consistent with previous studies showing that the capsule can impede factors critical for colonization [24,25]. The Newman sortase and *clfA* mutants each showed impaired fitness in the mouse gut. In contrast, strains with mutations in *clfB*, *spa*, *sdrCDE*, or the *ica* locus showed no fitness defect in the GI colonization model. This suggests that ClfA and perhaps other wall-anchored protein adhesins (possibly masked by the capsule) play a role in promoting *S*. *aureus* colonization of the GI tract. Competition experiments are commonly used to assess microbial fitness for colonization of the GI tract [19–21], probably because they are more sensitive and may reveal more subtle competitive advantages demonstrated by a particular wild type or mutant strain.

More striking, however, was our observation that *tagO* mutants of strains SA113 and Newman that fail to produce WTA were unable to colonize the nose or the GI tract of mice. This colonization defect was evident in groups of conventional mice inoculated with pure cultures of the mutant strain; competition assays were not necessary to demonstrate this impaired colonization phenotype in vivo. *S*. *aureus* mutants that lacked the D-ala or GlcNAc substituents of WTA, on the other hand, showed no defects in GI colonization. These findings suggest that the glycopolymer itself, and not its modifying groups, are critical for interactions with the host.

In vitro assays demonstrated that both the *tagO* and *clfA* mutants of strain Newman were less adherent to T84 cells in vitro than the parental strain Newman. These data suggest a critical role for these surface antigens in GI colonization. Baur et al. [33] recently reported that *S*. *aureus* WTA interacts with a scavenger receptor (SREC-I; scavenger receptor expressed by endothelial cell-I) detected on nasal epithelial cells to promote bacterial adherence. Whether such a receptor is found on intestinal epithelial cells remains to be determined.

In addition to its adherence defects, the *tagO* mutant showed fitness defects in transit through the mouse stomach and intestines. In an in vivo competition study, the *tagO* mutant was outcompeted by the WT strain within 1 h after inoculation. This finding is consistent with our in vitro observations that the *tagO* mutant was more susceptible than the parental strain to bile acids, proteases, and alpha defensin 5, which would be encountered in the GI tract. Like humans, mice have Paneth cells in the small intestinal crypts, and these cells secrete alpha defensins that show bactericidal activity against a number of microbes, including *S*. *aureus* [34].

*S*. *aureus* autolysins are activated by detergents (Triton X-100 and bile acids such as deoxycholate), defensins, and proteases [30]. Because these factors are present in the GI tract, we postulated that the Newman *tagO* mutant did not survive well because it is more susceptible to stress-induced autolysis than the wild-type strain. To address this, we created an *atl* deficient mutant of strain Newman. Atl is the major *S*. *aureus* autolytic enzyme, and it binds preferentially at the septum site (where WTA is less abundant) to facilitate cell division. However, Atl loses its selective localization to division sites in a *tagO* mutant. In the absence of WTA, Atl binding was reported to be evenly distributed on the cell surface, which explains the increased fragility and susceptibility of the Δ*tagO* mutant to autolysis [31,35]. As expected, the *atl* and the *atl tagO* double mutant were both resistant to Triton X-100-induced lysis (activation of endogenous autolysins), whereas both the *tagO* and *atl tagO* double mutant were highly susceptible to exogenous autolysins (in Newman culture supernatants).

Our final in vivo colonization studies showed that the WT strain Newman and its isogenic atl mutant were both proficient for GI colonization. However, neither the *tagO* nor the *atl tagO* double mutant colonized the mouse gut. These results indicate that the enhanced sensitivity of the *tagO* mutant to antimicrobial factors in the GI tract, as well as its poor adherence characteristics, likely contribute to its impaired fitness in vivo. However, the enhanced vulnerability of the *tagO* mutant to clearance from the GI tract is apparently not due to activation of its endogenous autolysins by host-induced bacterial stress. Previous studies have characterized the in vitro susceptibility of *S*. *aureus* Δ*tagO* to a variety of host immune defenses. Compared to the parental strains, a Δ*tagO* mutant showed increased susceptibility to antimicrobial fatty acids on human skin [36], but it was not more susceptible to killing by neutrophils, lysozyme [37], lactoferrin, or cationic antimicrobial peptides such as hNP1-3, LL37, or magainin II amide. Mutants lacking WTA were more resistant to mammalian group IIA phospholipase A_2_ and human β-defensin 3 (HBD-3) than the WT strain [29]. Additional studies will be necessary to further delineate the essential role of WTA in *S*. *aureus* colonization of the mammalian GI tract.

The murine colonization model may be useful in further characterization of factors that impact *S*. *aureus* carriage in the GI tract, with the acknowledged limitation that the human intestinal tract and its flora have characteristics distinct from those of the mouse [38]. Nonetheless, our results show that *S*. *aureus* WTA contributes to staphylococcal fitness within the GI tract, providing resistance to host bactericidal factors and promoting bacterial adherence to epithelial cells. A better understanding of mechanisms that lead to asymptomatic colonization by *S*. *aureus* may lead to preventive therapies that impact transmission among humans. Recent efforts to develop antimicrobials that target WTA [39–41] may lead to effective agents that diminish both nasal and GI colonization by MRSA and may impact invasive disease [23].

## Materials and Methods

### Bacterial strains

The *S*. *aureus* strains tested in the murine gastrointestinal colonization model are listed in Table 3; each was resistant to Sm. Mutations in the *S*. *aureus ica* locus, *spa*, and *atl* were moved into Sm-resistant Newman by transduction with phage 80α or phage 85 from the original antibiotic marked mutant strains [42–44]. The *tagO*::*tet* mutation was moved from *S*. *aureus* RN4220 into Newman *Δatl* as described previously [22]. Mutants were confirmed by PCR or Southern blot analysis and were phenotypically identical to the parental strains in terms of the growth rate, hemolysis on sheep blood agar plates, and the metabolic profile on API Staph test strips (Biomerieux, Inc., Durham, NC). *S*. *aureus* strains were cultivated in TSB to the logarithmic growth phase, unless otherwise noted. Sm (0.5 mg/ml; Sigma Chemical Co., St. Louis, Mo.), erythromycin (Em; 5 μg/ml; Sigma), tetracycline (Tc; 2.5 μg/ml; Sigma), or spectinomycin (Spc; 100 μg/ml; MP Biomedicals, Solon, OH) was added to culture medium for selection where appropriate.

**Table 3:** Sm-resistant *S*. *aureus* strains used in this study.

*S. aureus* strain	Relevant characteristics ^a^ ^,^ ^b^	Reference
Newman	WT, capsule type 5	[12]
Newman Δ*atl*	Lacks the major autolysin, Em^r^	This study
Newman Δ*clfA*	Lacks clumping factor A; clfA1::Tn*917*	[52]
Newman Δ*cap5G*	Lacks type 5 capsule production, Em^r^	[53]
Newman Δ*clfB*	Lacks clumping factor B; Em^r^	[12]
Newman Δ*dltA*	Lacks teichoic acid D-alanine esters, Spc^r^	[22]
Newman Δ*ica*	Lacks poly-N-acetyl glucosamine, Tc^r^	This study
Newman Δ*spa*	Lacks protein A	[44]
Newman Δ*srtA*	Lacks sortase A; adhesin-deficient; Em^r^	[12]
Newman Δ*tagO*	Lacks wall teichoic acid, Tc^r^	[22]
Newman Δ*tagO* (pRB*tagO*)	Complemented Δ*tagO*; restored for WTA production	[22]
Newman Δ*atl*Δ*tagO*	Lacks autolysin and wall techoic acid; Tc^r^, Em^r^	This study
NCTC 8325–4	NCTC 8325 cured of three prophages	[54]
NCTC 8325–4 *hlb*::Φ42E	NCTC 8325–4 Φ42E lysogen, beta toxin negative	[55]
NCTC 8325–4 Δ*atl*	Lacks the major autolysin, Em^r^	[43]
RN4220 Δ*tarM* Δ*tarS*	Lacks β-*O*-GlcNAc and β-*O*-GlcNAc groups on WTA	[56]
SA113	WT, acapsular	[57]
SA113 Δ*tagO*	Lacks wall teichoic acid, Em^r^	[13]
SA113 Δ*dltA*	Lacks teichoic acid D-alanine esters, Spc^r^	[58]

^a^ Mutant strains were derived from Sm-resistant WT strain.

^b^ Tc, tetracycline; Spc, spectinomycin; Em, erythromycin.

### Ethics statement

Animal experiments were approved by the Longwood Medical Area's Institutional Animal Care and Use Committee under protocol 86–02131. All studies were performed in strict accordance with the National Institutes of Health standards as set forth in "Guide for the Care and Use of Laboratory Animals" (DHSS Publication No. (NIH) 85–23).

### Murine model of gastrointestinal colonization

Conventional female ICR mice aged 4–6 wks old were purchased from Harlan Sprague Dawley, Inc. (Indianapolis, IN) or Charles River Laboratories (Wilmington, MA). The mice were given drinking water containing Sm (0.5 g/L) one day prior to inoculation and for the course of experiment (unless otherwise noted), and the drinking water and cages were changed twice a week. Conventional mice were inoculated without prior anesthesia by the intranasal route with 10 μl of an *S*. *aureus* suspension as described [12]. Gastrointestinal colonization by *S*. *aureus* was evaluated by quantitative cultures of representative mouse stool pellets that were collected prior to inoculation and weekly thereafter. The samples were weighed, suspended in 2–5 ml of TSB, diluted, and plated quantitatively on mannitol salt agar (MSA) containing 0.5 mg/ml Sm. The plates were incubated aerobically for 48 h at 37°C, the colonies were enumerated, and the data were expressed as CFU/g stool. Significant (*P* < 0.05) differences between quantitative culture results were determined by the two-tailed Student t test, and the Welch correction was applied to pairs with unequal variances. *P*-values for experiments having three or more groups were determined by the Kruskal-Wallis test with Dunn’s multiple comparison analysis (Prism; GraphPad Software, La Jolla, CA).

Germfree Swiss-Webster female mice (3–4 weeks old) were purchased from Taconic Farms (Hudson, NY) and maintained in a negative-pressure BL2 isolator by personnel at the gnotobiotic core facility of the Harvard Digestive Diseases Center. *S*. *aureus* suspensions were drawn into a sterile syringe with a neonatal feeding tube, and ~ 200 μl (~10^9^ CFU) was orally fed to the mice. Gnotobiotic mice were given sterile (no Sm) drinking water, and their stools were cultured on nonselective medium (tryptic soy agar [TSA] plates).

For competition experiments, the mutant and parental strains were mixed in equal numbers (total 10^9^ CFU/mouse) prior to inoculation of conventional mice. The input CFU ratio was calculated by dividing the wild type inoculum CFU by the mutant inoculum CFU (WT/mutant). At various time points, stool samples were collected and plated quantitatively on MSA + Sm plates. Colonies of the mutant strains were distinguished from those of the parental strain by transferring (via sterile toothpicks) ~100 colonies to TSA and TSA + antibiotic (Tc, Em, or Spc) plates. The output CFU ratio was determined by dividing the WT CFU by the mutant CFU for each fecal sample. A competitive index (CI) was calculated as described [45] by dividing the output ratio by the input ratio, and the CI was expressed as log_10_. A CI = 0 indicates similar numbers of CFU of the two competing strains recovered in vivo, suggesting comparable fitness of the strains in vivo. A CI >1 indicates the fitness advantage of the parental strain over the mutant, whereas a CI <1 indicates that the mutant is more fit than the parental strain. A Wilcoxon signed-rank test was used to determine whether the CI values were significantly different from the hypothetical value of zero. For in vitro competition experiments, the parental and mutant strains were inoculated together into 5 ml of TSB, grown with aeration for either 2 or 24 h at 37°C, and subsequently plated quantitatively. Colonies of the Δ*tagO* mutant were distinguished from those of the parental strain by their antibiotic resistance as described above, and the CI was calculated.

### Competition between Newman and Newman Δ*tagO* in transit through the GI tract

To determine whether the *tagO* mutant survived as well as the parental strain in its transit through the mouse stomach, rodent chow was withheld for 3 h prior to inoculation of mice with either *S*. *aureus* Newman, Newman Δ*tagO*, or Newman Δ*tagO* Δ*atl*. The mice were euthanized by CO_2_ asphyxiation after 1 h, and quantitative cultures were performed on homogenates made from different segments of the GI tract. However, variations in the recovery of *S*. *aureus* among individual mice were substantial, and we were unable to draw meaningful conclusions from these data. To circumvent this problem, competition experiments were performed as described above in which equal numbers (~5 x 10^8^ CFU) of the two strains were mixed before inoculation. One hour after bacterial challenge, the mice were euthanized, the GI tract was excised, and the stomach, small intestine (4 segments), cecum, and colon were separated. Each segment was weighed, homogenized in 1 ml TSB, and cultured quantitatively on MSA + Sm plates. Colonies of the Δ*tagO* mutant were distinguished from those of the parental strain by replica plating on TSA + Tc or TSA + Em, and the CI was calculated.

### Adherence of Newman vs. *ΔtagO* to T84 cells

Polarized monolayers of human intestinal T84 cells were cultured on 0.33 cm^2^ polyester Transwell inserts (Corning, Acton, MA) as previously described [46]. Log-phase TSB cultures of *S*. *aureus* Newman or its isogenic mutants were washed in Hanks Balanced Salt Solution (HBSS) and incubated at a multiplicity of infection of 100 on the apical surfaces of polarized T84 monolayers for 1 h at 37°C. Inserts were washed five times with HBSS and processed for confocal microscopy as previously described [47]. Briefly, the T84 cell monolayers were fixed in 4% paraformaldehyde for 20 min. The cells were washed 3x in HBSS, permeabilized with 0.2% Saponin/HBSS solution, washed again, and blocked with 10% normal goat serum (NGS)/HBSS solution. Immunostaining was performed with mouse anti-ZO-1 (1:200; Invitrogen) and *S*. *aureus* rabbit antiserum (raised to whole killed bacteria and diluted 1:400) in 10% NGS/HBSS. The cells were incubated with AlexaFluor 568 labeled goat anti-mouse conjugate and AlexaFluor 488 labeled goat anti-rabbit conjugate (both 1:400 in 10% NGS/HBSS solution; Invitrogen) for 1 h at room temperature. Samples were imaged by confocal microscopy on a Nikon TE2000 inverted microscope (Nikon Instruments, Melville, NY) coupled to a Perkin-Elmer spinning disk confocal unit (Boston, MA), using a Nikon PlanFluor 40× (1.3 NA) and Nikon PlanApo 60x (1.4 NA) oil immersion objective lens and an Orca AG scientific-grade cooled CCD camera (Hamamatsu Photonics K.K., Japan). Confocal images, collected en face to the apical membrane, were 3D stacks collapsed into a single projection. Adherence was quantified by counting the total bacteria in 20 random fields of view (FOV) among triplicate samples with a 63X objective and a 10X ocular lens.

### Bactericidal assays

Newman WT and Δ*tagO* were cultivated in TSB to log phase at 37°C, washed in 10 mM phosphate buffer (pH 7.4), and suspended to 1–3 x 10^8^ CFU/ml in either buffer, bile salts (sodium deoxycholate [DOC]; Sigma), or human α-defensin 5 (HD5) (Peptide Institute, Inc., Osaka, Japan). Susceptibility to low pH was assessed by suspending *S*. *aureus* strains in glycine buffer (pH 3.0, 3.3, or 3.6) or in phosphate buffer at neutral pH. Bacterial suspensions were incubated at 37°C for 1 to 2 h, and viable counts were determined. Susceptibility to proteases was assessed by preparing serial dilutions in optimal buffers (proteinase K in 10 mM phosphate buffer, pH 7.4; pepsin in glycine buffer, pH 3.6; trypsin in 10 mM phosphate, pH 8.0 buffer). The protease solutions were then inoculated with logarithmic-phase *S*. *aureus* to a final concentration of 1 x 10^6^ CFU/ml, and viability counts were performed after incubation for 24 h at 37°C.

The bactericidal activity of *S*. *aureus* culture supernatants was assessed by culturing the *S*. *aureus* strains in TSB at 37°C for ~21 h. Filter-sterilized culture supernatants were inoculated to a final 10^5^ CFU/ml with strain Newman or Δ*tagO*, Δ*tagO* (pRB*tagO*) [22], Δ*atl*, or Δ*atl* Δ*tagO* mutants. CFU/ml determinations were performed after incubation for 1 to 5 h at 37°C. For some assays, the Newman supernatant was boiled for 5 min, treated with a protease inhibitor cocktail (Sigma P-8465), or supplemented with 0 to 4 mg peptidoglycan purified from *S*. *aureus* Lafferty [48] as described [22] before inoculation with strain Newman or its *tagO* mutant.

### *S*. *aureus* autolysin activity

Triton X-100-induced autolysin assays were performed as described by Shaw et al. [49]. Briefly, Newman WT and Δ*tagO* strains were grown to logarithmic phase, and the bacterial cells were washed twice and suspended to OD_650 nm_ of 1.0 in 50 mM Tris-HCl (pH 7.6), 2 mM CaCl_2_, 0.05% Triton X-100. The suspensions were incubated in a 37°C shaking water bath, and the OD_580 nm_ was monitored for 3 h.

Zymography was used to detect the lytic activities in culture supernatants from Newman and Newman Δ*tagO*, as described by Groicher et al. [50]. Overnight culture supernatants, concentrated with Centricon-5 centrifugal filter units (Millipore) to a protein concentration of 2.65 mg/ml, were electrophoresed in a 10% polyacrylamide SDS gel supplemented with 1 mg/ml *S*. *aureus* cell walls. After washing the gel in 1% Triton X-100 and 25 mM Tris-HCl buffer overnight to allow cell wall hydrolysis, the gel was stained with 1% methylene blue and destained in water. Lytic zones in the gel indicated proteins with peptidoglycan hydrolase activity.

*S*. *aureus* autolysin extracts were prepared by 3 M LiCl treatment as described [51]. Peptidoglycan samples, prepared from strains Newman or Newman Δ*tagO* as described previously [22], were suspended in 0.01 M KHPO_4_ buffer (pH 7.1) to OD_580 nm_ of 0.5 to 0.6. The suspensions were mixed with an *S*. *aureus* Newman autolysin extract (25 μg/ml protein), incubated in a 30°C water bath, and OD_580 nm_ readings were taken at 1 h intervals. Lytic activity was expressed as a percentage of the OD_580 nm_ at time zero.

## Supporting Information

S1 Fig *S*. *aureus* GI colonization is dependent upon the addition of Sm to the drinking water. Mice received drinking water supplemented with Sm for the entire 3-week period, for only one week, or not at all. Each dot indicates the CFU *S*. *aureus*/g stool for a single mouse, and the median of each group of animals is indicated by a horizontal line. The lower limit of detection by culture was 2.5 log CFU/g stool. *P*-values were determined by Kruskal-Wallis test with Dunn’s multiple comparison test. * *P* < 0.05; ** *P* < 0.01.
(TIF)

S2 Fig An *S*. *aureus clfA* mutant showed a competitive defect in colonization of the murine GI tract following inoculation by oral gavage. The competitive index (CI) was defined as the log_10_ output ratio/ input ratio. A CI <0 indicates a mutant with a colonization advantage over the WT strain, and a CI >0 indicates a mutant with a colonization disadvantage compared to the WT. Horizontal bars represent the median competitive index for groups of 10 mice. *P*-values were determined by the Wilcoxon signed-rank test. *P < 0.05; ** *P* < 0.01.
(TIFF)

S3 Fig Newman Δ*ica*, Newman Δ*clfB*, Newman Δ*spa*, and Newman Δ*sdrCDE* showed no colonization defect. (TIFF)

S4 Fig Isogenic mutants lacking the serotype 5 capsule (CP), sortase (SrtA), ClfA, or beta hemolysin (Hlb) colonized individual groups of mice at levels comparable to those of the parental strain. (TIFF)

S5 Fig *S*. *aureus* Newman Δ*tagO* and Δ*atl* Δ*tagO* showed impaired GI colonization following inoculation by oral gavage with 10^9^ CFU. Fecal pellets were cultured quantitatively at the indicated time points. Each dot indicates the CFU *S*. *aureus*/g stool for a single mouse, and the median of each group of animals is indicated by a horizontal line. The lower limit of detection by culture was ~2 log CFU/g stool.
(TIFF)

S6 Fig A *tarM tarS* double mutant of *S*. *aureus* RN4220, which lacks alpha- and beta-*O*-linked GlcNAc modifications of its WTA, shows no defect in GI colonization over a 21 day time period. The mice were inoculated by oral gavage with 10^9^ CFU of each *S*. *aureus* strain, and fecal pellets were cultured quantitatively at indicated time points. Each dot indicates the CFU *S*. *aureus*/g stool for a single mouse, and the median of each group of animals is indicated by a horizontal line. The lower limit of detection by culture was ~2.5 log CFU/g stool.
(TIFF)

S7 Fig Both the WT *S*. *aureus* strain Newman and the *tagO* mutant were killed under low pH conditions (glycine buffer, pH 3.0–3.6). (TIF)

S8 Fig Newman Δ*tagO* was more sensitive than the parental strain to overnight treatments with the proteolytic enzymes pepsin and trypsin. (TIFF)

S9 Fig Compared to the control *S*. *aureus* strain V8, strain Newman was only weakly proteolytic, and no significant differences were observed between the protease activity of culture supernatants derived from the WT or the Δ*tagO* mutant. (TIFF)

S10 Fig Autolysin extracts from strain Newman and Newman Δ*tagO* showed similar lytic activity toward peptidoglycan prepared from the wild type *S*. *aureus* strain Lafferty. (TIFF)

S11 Fig The Newman WT and *atl* mutant both colonized the mouse GI tract, whereas the Δ*tagO* and the Δ*atl* Δ*tagO* double mutant were not recovered from the mouse stools 7 and 14 days after inoculation. Each dot indicates the CFU *S*. *aureus*/g stool for a single mouse, and the median of each group of animals is indicated by a horizontal line. The lower limit of detection by culture was ~200 CFU/g stool. *P*-values were determined by Kruskal-Wallis test with Dunn’s multiple comparison test. * *P* < 0.05; ** *P* < 0.01.
(TIFF)
