# Supplementary figures and images for: Staphylococcus aureus Colonization of the Mouse Gastrointestinal Tract Is Modulated by Wall Teichoic Acid, Capsule, and Surface Proteins
Source: PLoS Pathog. 2015 Jul 22;11(7):e1005061. doi: 10.1371/journal.ppat.1005061 (PMC4511793; doi:10.1371/journal.ppat.1005061)

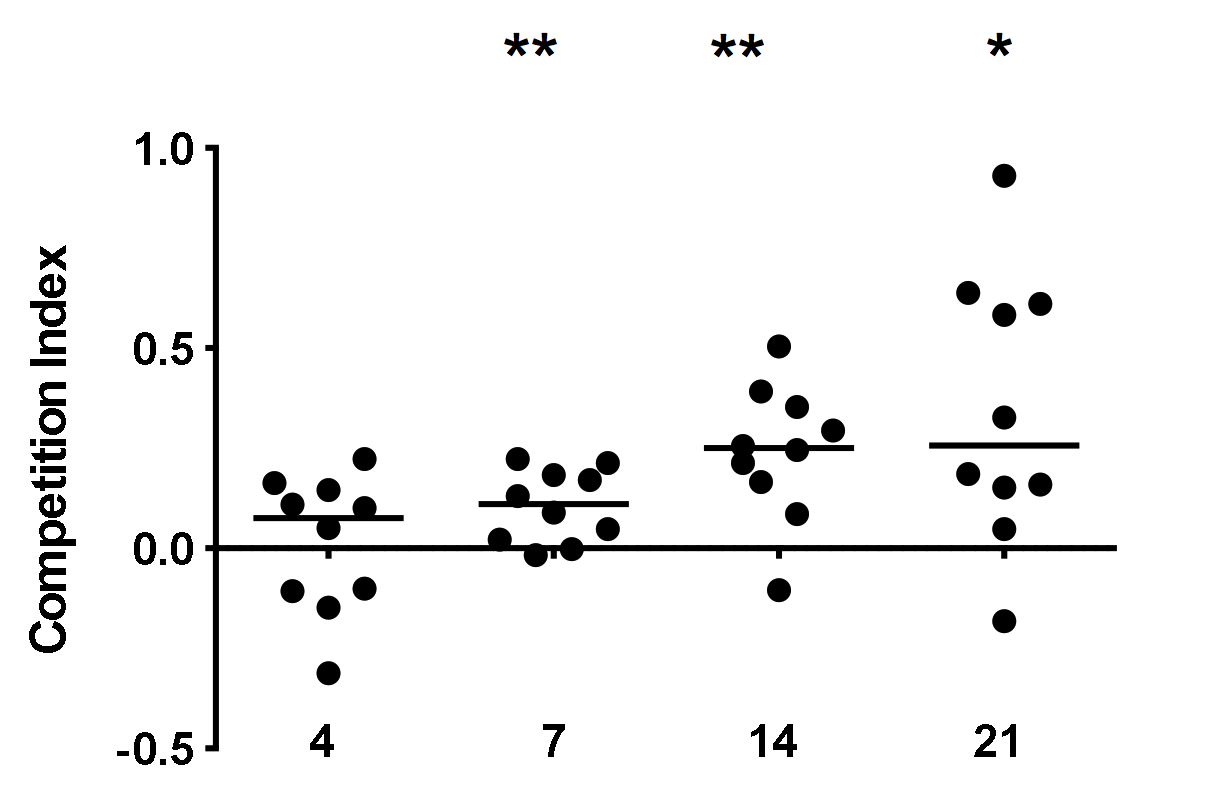

Supplement: S2 Fig — The competitive index (CI) was defined as the log10 output ratio/ input ratio. A CI <0 indicates a mutant with a colonization advantage over the WT strain, and a CI >0 indicates a mutant with a colonization disadvantage compared to the WT. Horizontal bars represent the median competitive index for groups of 10 mice. P-values were determined by the Wilcoxon signed-rank test. *P < 0.05; ** P < 0.01. (TIFF) [file ppat.1005061.s002.tiff]

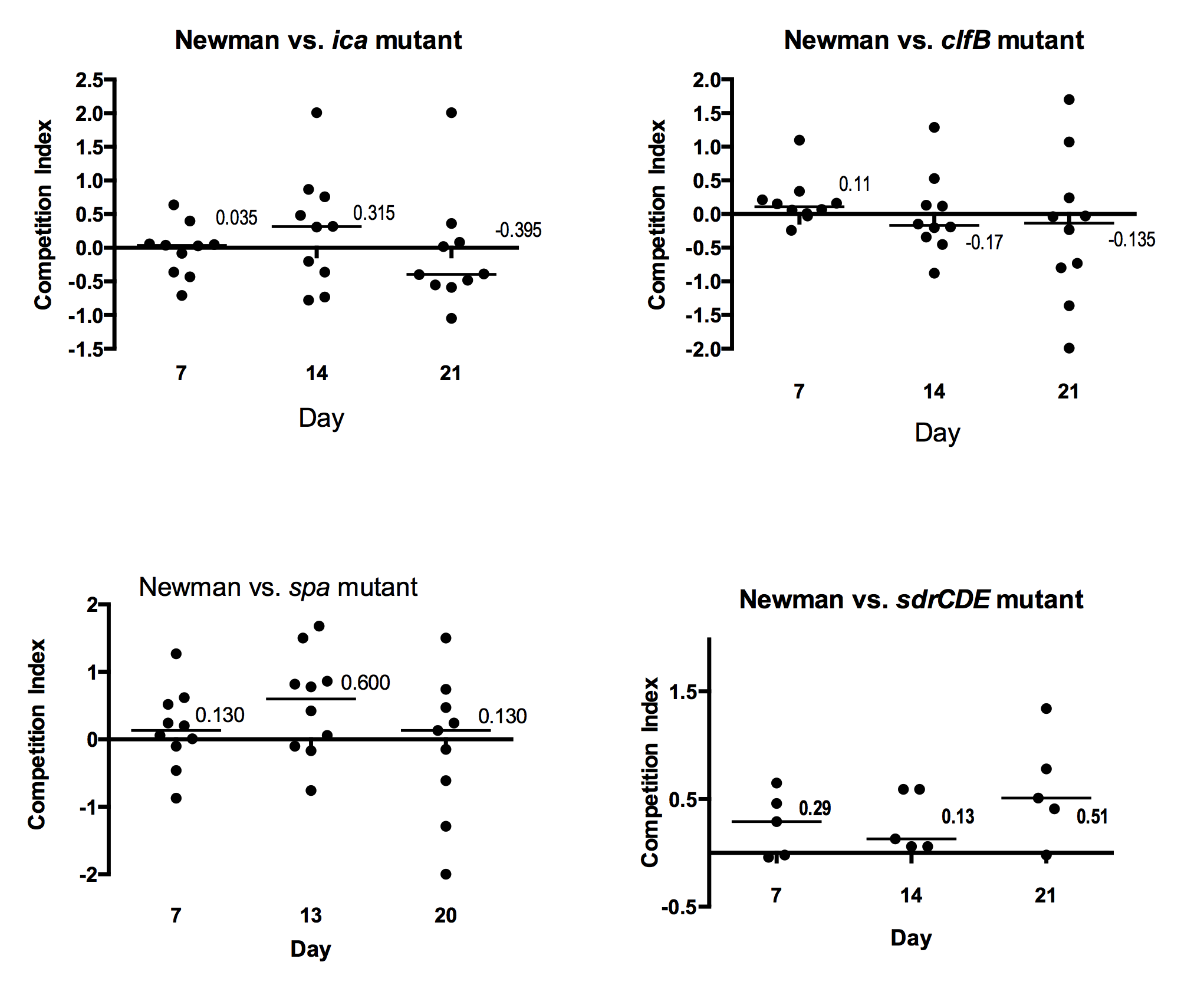

Supplement: S3 Fig — (TIFF) [file ppat.1005061.s003.tiff]

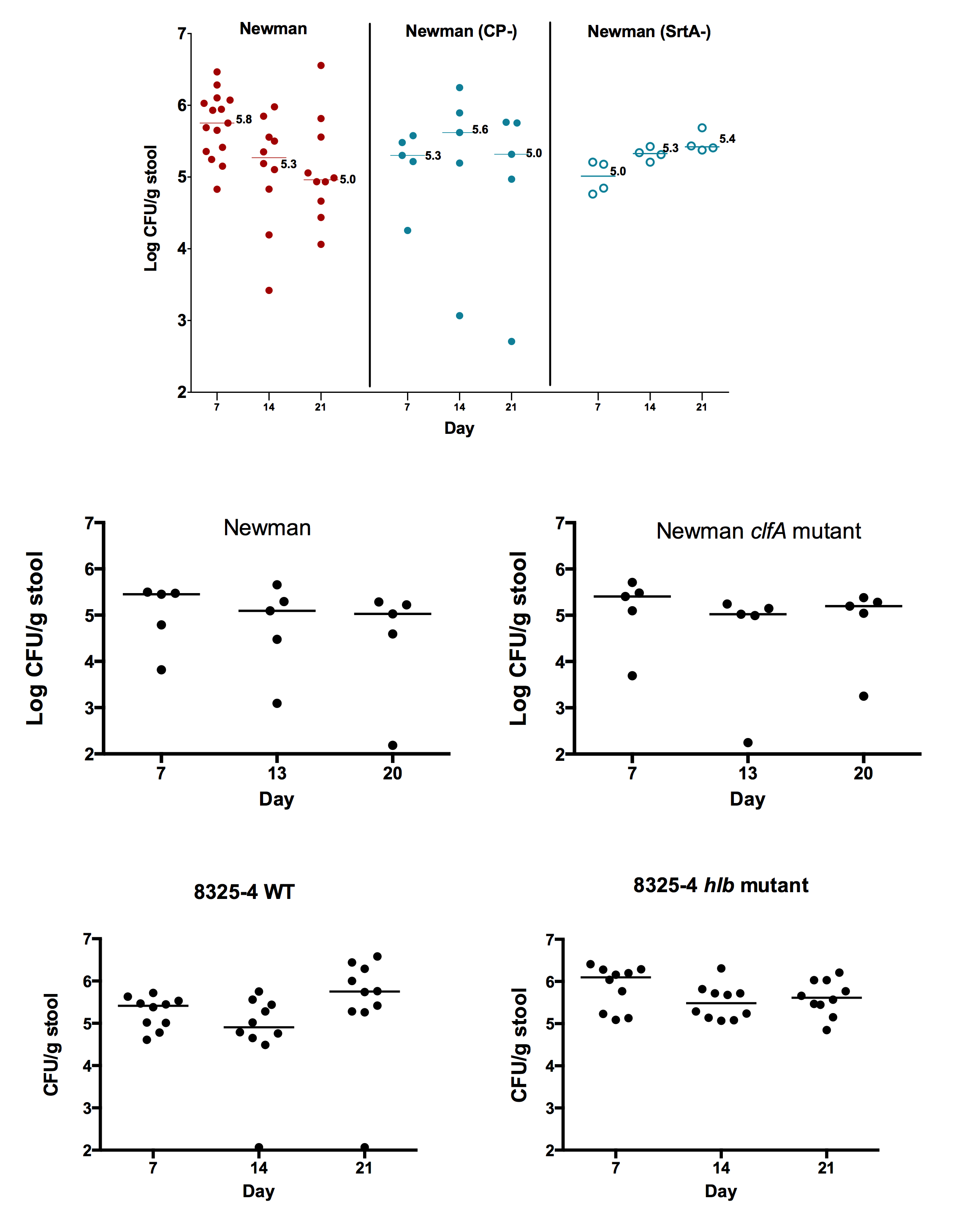

Supplement: S4 Fig — (TIFF) [file ppat.1005061.s004.tiff]

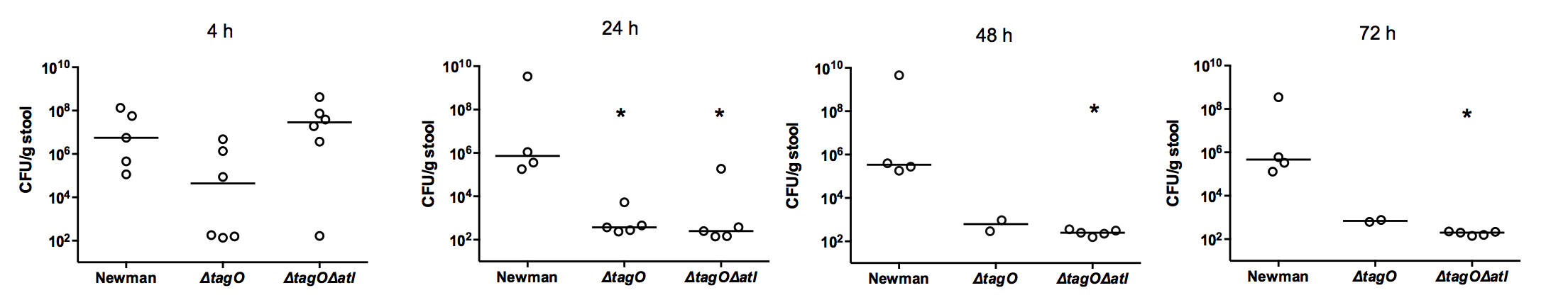

Supplement: S5 Fig — Fecal pellets were cultured quantitatively at the indicated time points. Each dot indicates the CFU S. aureus/g stool for a single mouse, and the median of each group of animals is indicated by a horizontal line. The lower limit of detection by culture was ~2 log CFU/g stool. (TIFF) [file ppat.1005061.s005.tiff]

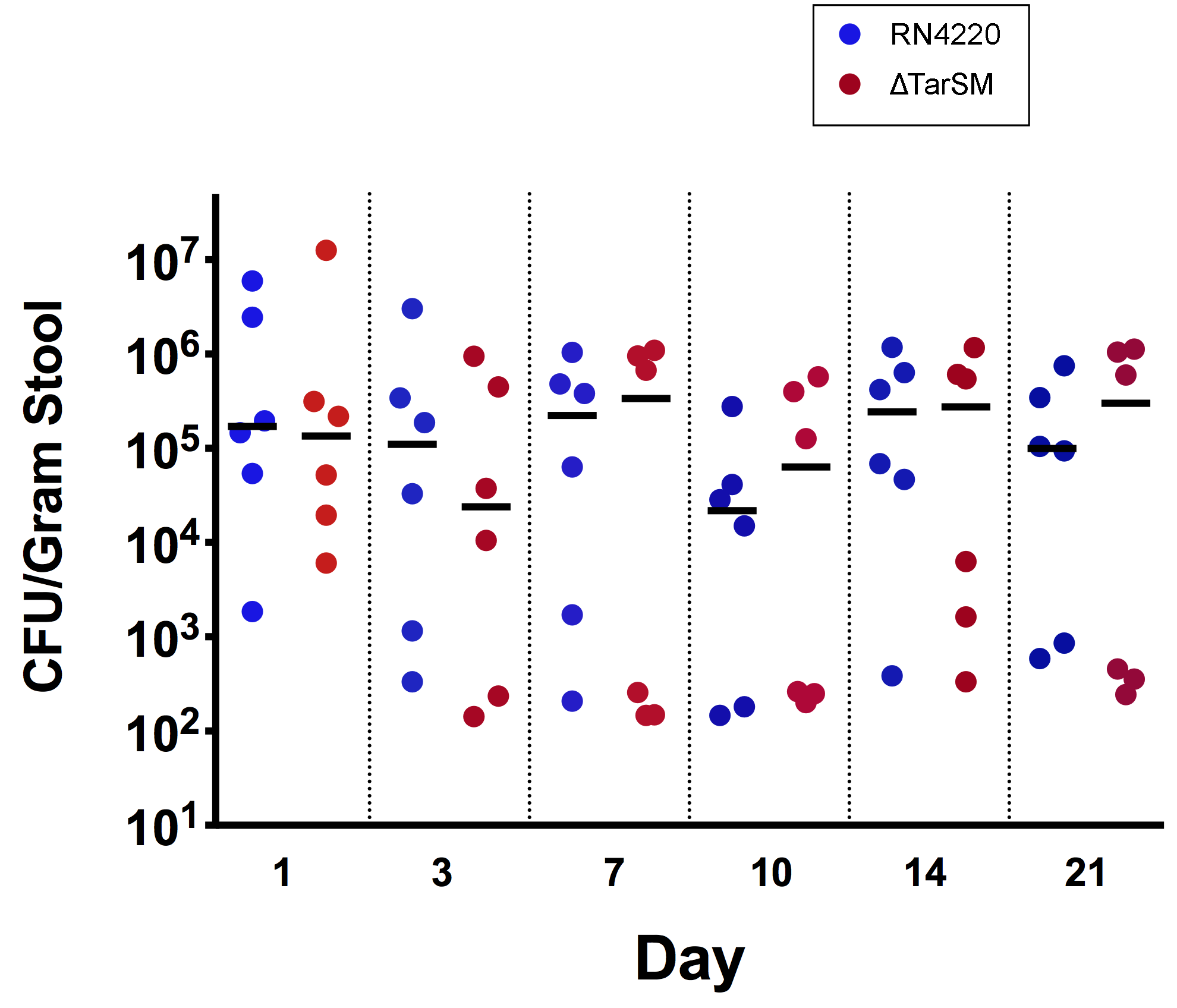

Supplement: S6 Fig — The mice were inoculated by oral gavage with 109 CFU of each S. aureus strain, and fecal pellets were cultured quantitatively at indicated time points. Each dot indicates the CFU S. aureus/g stool for a single mouse, and the median of each group of animals is indicated by a horizontal line. The lower limit of detection by culture was ~2.5 log CFU/g stool. (TIFF) [file ppat.1005061.s006.tiff]

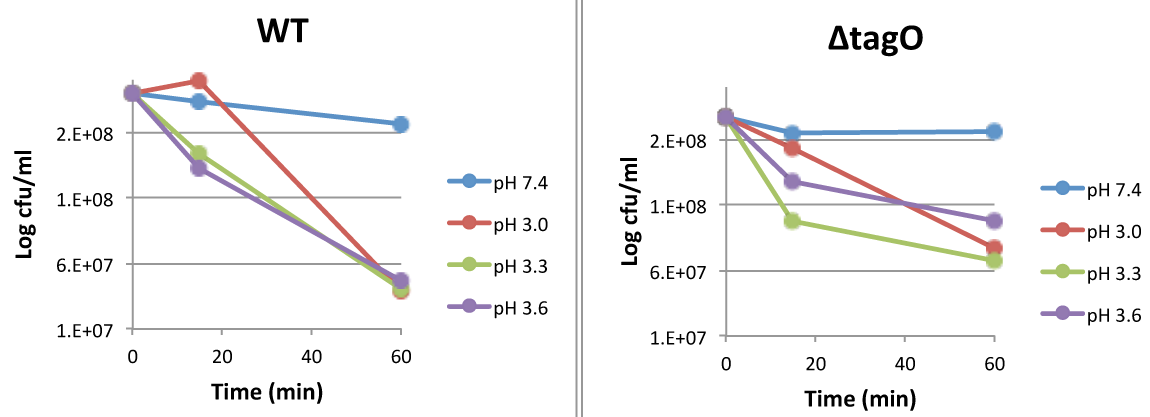

Supplement: S7 Fig — (TIF) [file ppat.1005061.s007.tif]

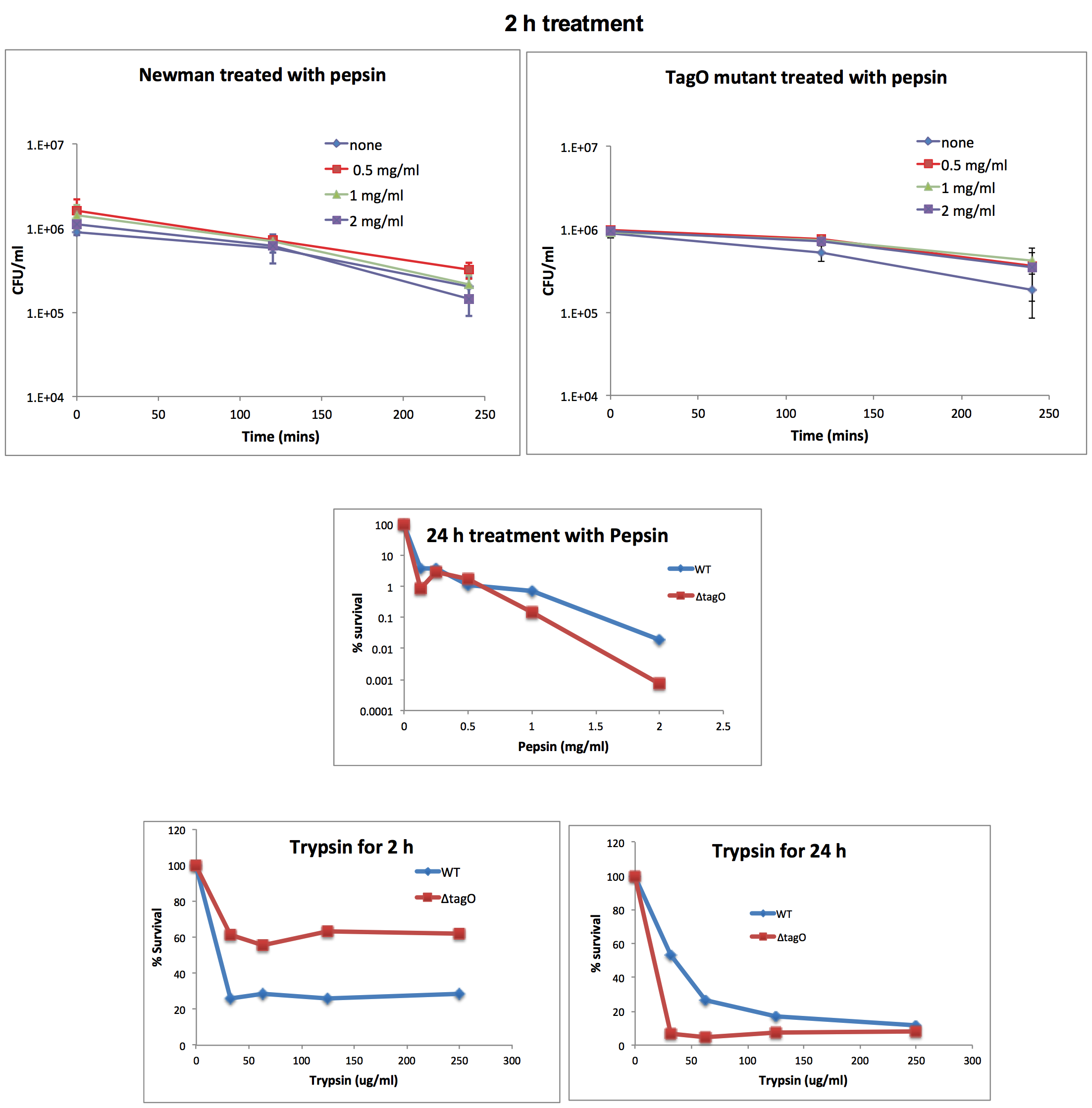

Supplement: S8 Fig — (TIFF) [file ppat.1005061.s008.tiff]

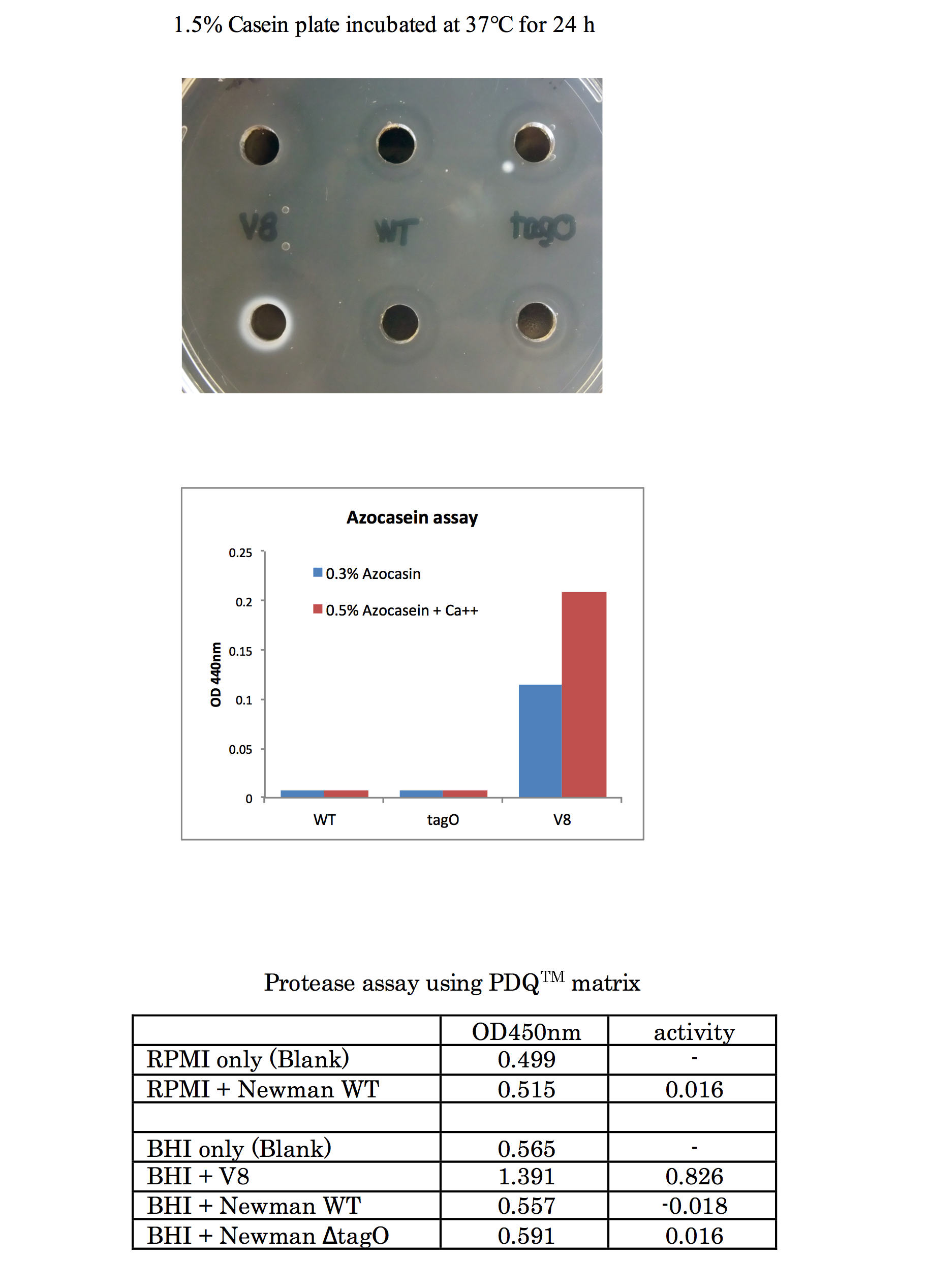

Supplement: S9 Fig — (TIFF) [file ppat.1005061.s009.tiff]

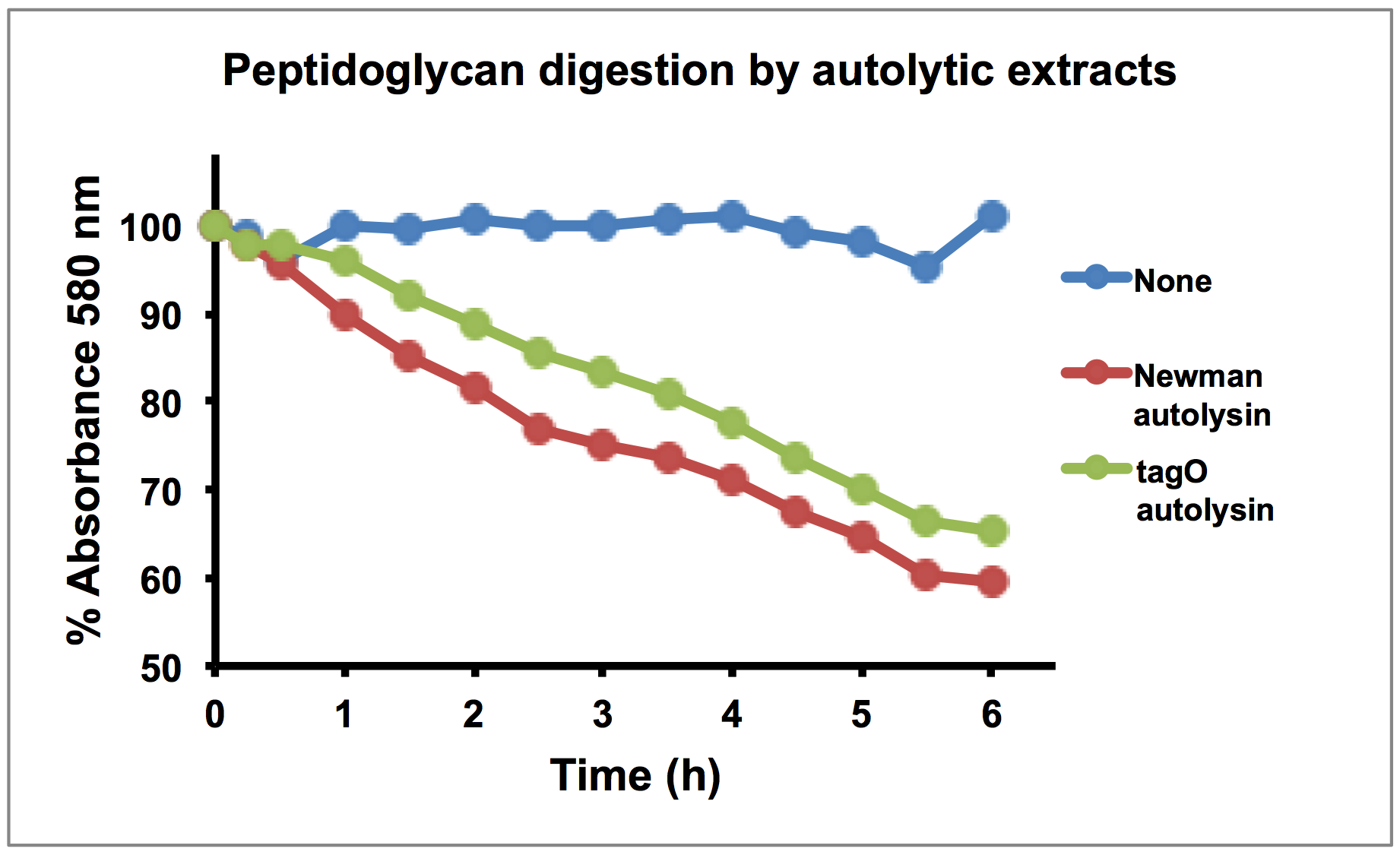

Supplement: S10 Fig — (TIFF) [file ppat.1005061.s010.tiff]

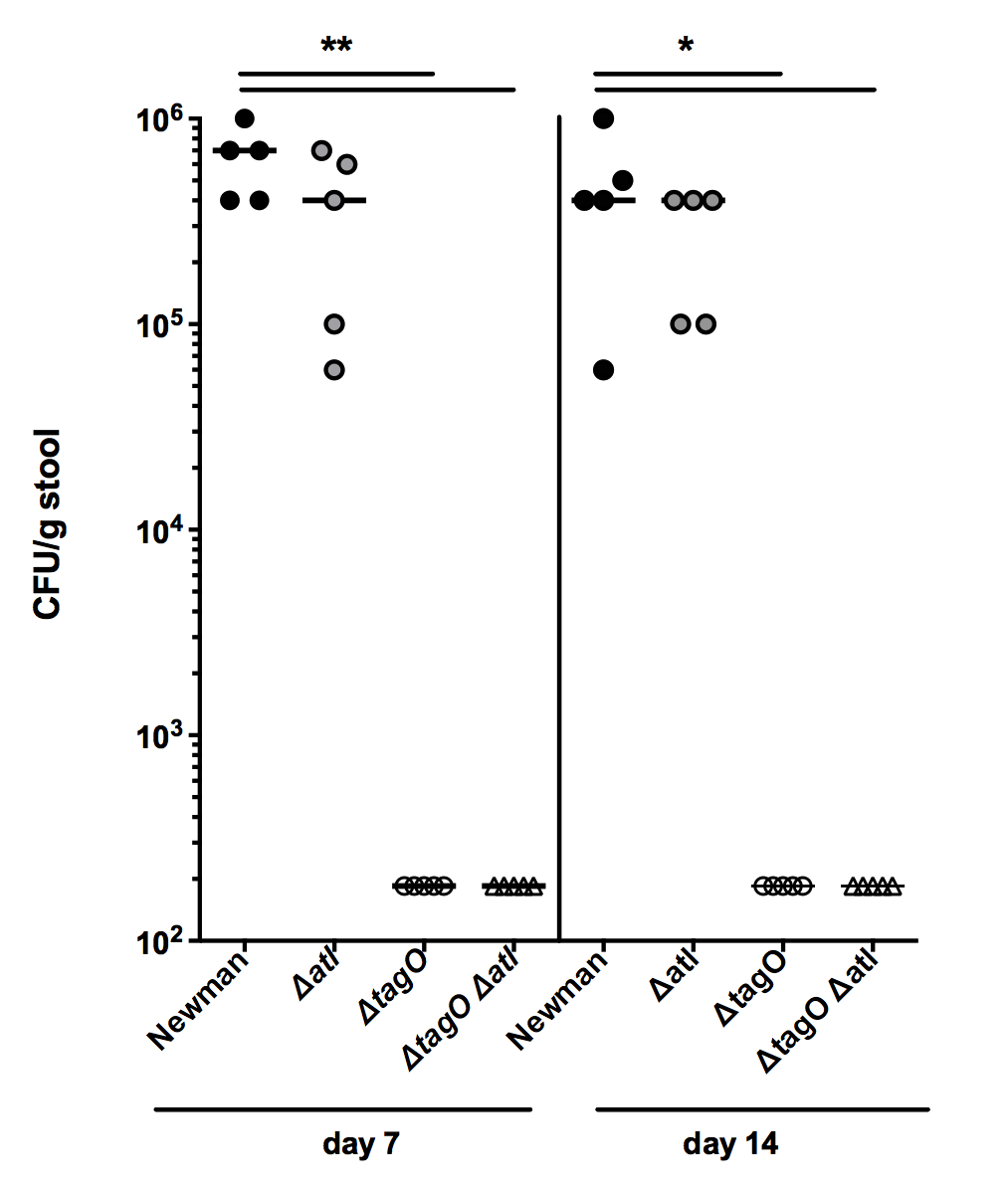

Supplement: S11 Fig — Each dot indicates the CFU S. aureus/g stool for a single mouse, and the median of each group of animals is indicated by a horizontal line. The lower limit of detection by culture was ~200 CFU/g stool. P-values were determined by Kruskal-Wallis test with Dunn’s multiple comparison test. * P < 0.05; ** P < 0.01. (TIFF) [file ppat.1005061.s011.tiff]
